# Tensile characterization and life cycle assessment of fungal-colonized 3D-printed PLA/wood biocomposites

**DOI:** 10.1038/s41598-026-65923-5

**Published:** 2026-08-22

**Authors:** Narges Panjalipoursangari, Yanlong Zhu, Wolfgang H. Müller, Christina Völlmecke

**Affiliations:** 1 Continuum Mechanics and Materials Theory Group, Technical University of Berlin, Einsteinufer 5, 10587 Berlin, Germany; 2 Stability and Failure of Functionally Optimized Structures Group, Technical University of Berlin, Einsteinufer 5, 10587 Berlin, Germany

**Keywords:** PLA biocomposites, *Fomes fomentarius*, Wood-particle reinforcement, Mycelium colonization, Sustainable materials, Material extrusion additive manufacturing (MEX AM), Engineering, Materials science

## Abstract

This study investigates the mechanical behaviour of additively manufactured biocomposites based on PolyLactic Acid (PLA) reinforced with nominal wood-particle contents ranging from 10 to 50wt. % and subsequently subjected to controlled growth of the fungus *Fomes fomentarius*. The aim was to analyse how post-printing fungal colonisation and the associated processing conditions influence the mechanical characteristics of the resulting wood-containing PLA specimens, particularly Young’s modulus (*E*), mean Ultimate Tensile Strength (UTS), and overall deformation behaviour. Standardised tensile tests were performed, and the corresponding stress–strain curves were evaluated. The investigated PLA/wood material combinations exhibited noticeable differences in tensile behaviour, with mean Young’s modulus (*E*) values ranging from 2231 to 2685 $\mathrm {N/mm^2}$ and mean UTS values ranging from 31 to 38 $\mathrm {N/mm^2}$.  Specimens subjected to fungal colonisation and the associated incubation, drying, and handling procedure consistently exhibited lower mean *E* and mean UTS values than the corresponding untreated specimens  . Mean Young’s modulus decreased by approximately 7–11 %, while UTS decreased by approximately 2–7 %, depending on the material combination. Microscopic observations confirmed successful surface colonisation by *Fomes fomentarius*, whereas fungal growth within the internal specimen structure could not be detected. In addition, a supplementary screening-level Life Cycle Assessment (LCA) was conducted to compare the environmental impacts associated with the investigated PLA/wood material combinations during the Material Extrusion Additive Manufacturing (MEX AM) fabrication stage. The results indicate that electricity consumption dominated the Global Warming Potential (GWP), whereas material composition had a stronger influence on Acidification Potential (AP) and Eutrophication Potential (EP). The combined mechanical and screening-level environmental assessment provides indicative insights into the trade-offs associated with bio-based PLA/wood material combinations and their post-printing biological modification, supporting future work on more sustainable additively manufactured material systems.

## Introduction

Growing concerns regarding plastic pollution, fossil resource depletion, and climate change have intensified the search for sustainable alternatives to petroleum-based polymers. In this context, bio-based and biodegradable materials have gained increasing scientific and industrial attention due to their potential to reduce environmental impacts while maintaining functional performance in engineering applications. Among these materials, PolyLactic Acid (PLA) is one of the most widely investigated bio-based thermoplastics because of its renewable origin, biodegradability, and compatibility with conventional polymer-processing and additive manufacturing technologies^1,2^. PLA is commonly derived from renewable agricultural feedstocks such as corn starch or sugar cane and exhibits favourable properties including relatively high stiffness, dimensional stability, low processing temperatures, and good printability. These characteristics have contributed to its widespread use in Material Extrusion Additive Manufacturing (MEX AM), a three-dimensional 3D-printing technology that fabricates components layer-by-layer directly from digital geometries^3,4^. Consequently, PLA is frequently employed for lightweight structures, rapid prototyping, and sustainable manufacturing applications.

Despite these advantages, PLA also exhibits several limitations, including intrinsic brittleness, limited thermal resistance, and relatively low impact strength, which restrict its broader use in load-bearing and structural applications^3,4^. To overcome these limitations, the incorporation of natural lignocellulosic fillers has attracted increasing scientific interest. In particular, wood particles are considered promising bio-based fillers due to their low density, renewability, widespread availability, and low cost^5,6^. When incorporated into PLA filaments, wood particles form bio-based composites in which the lignocellulosic filler can influence stiffness, deformation behaviour, and printability depending on the particle characteristics, filler content, and interfacial adhesion between the wood particles and the PLA matrix^7,8^. The mechanical response of PLA/wood composites is influenced by several factors, including the nominal wood-particle content, filament formulation, processing conditions, additive manufacturing parameters, and the characteristics of the incorporated wood particles, such as their size and morphology^9–11^. Commercially available PLA/wood composite filaments have already been investigated with respect to their mechanical properties and processability in MEX AM^3,5,11^. However, little is known about how post-printing fungal colonisation and the associated processing conditions affect the tensile behaviour of these material combinations. Understanding this interaction is essential for the development of fungal-colonised additively manufactured bio-based composites.

More recently, mycelium-based materials have attracted increasing scientific attention as sustainable biological materials. Mycelium, the filamentous vegetative network of fungi, can grow on lignocellulosic substrates and form lightweight bio-based structures^12,13^. Although fungal colonisation is not primarily intended to improve the tensile strength of PLA/wood composites, it may provide additional functionalities that are not available in the untreated material. Mycelium can create biologically active or biodegradable surface layers, improve the integration of printed components into bio-based systems, and enable the development of hybrid living or biofabricated materials. Such approaches are increasingly being investigated for applications in sustainable construction, packaging, insulation, architectural components, and circular material systems. Before these applications can be realised, however, it is essential to understand how fungal colonisation influences the mechanical behaviour of the printed substrate. Due to their biological origin and cultivation-based fabrication process, mycelium materials are increasingly investigated for applications in packaging, architecture, thermal insulation, and biofabrication. In parallel with these developments, researchers have begun exploring the integration of fungal-based materials with additively manufactured structures. In recent approaches involving thermoplastic components, mycelium is generally not incorporated directly into the molten polymer during manufacturing. Instead, fungal colonisation is typically applied after fabrication onto previously manufactured structures^14–17^. In such systems, fungal growth may influence the surface condition and mechanical response of the fabricated components.

Although PLA/wood material combinations and mycelium-based materials have each been investigated individually, only limited research has addressed their combined use within additively manufactured hybrid material combinations. For example, Sharma and Le Ferrand^17^ recently reported mycelium-bound composites enabled by 3D-printed gyroid scaffolds, demonstrating the growing interest in combining additive manufacturing with fungal-based materials. However, their work focused on scaffold-supported mycelium composites, whereas the mechanical response of MEX-manufactured PLA/wood material combinations following fungal colonisation and the associated processing conditions remains largely unexplored. In particular, there is limited understanding of how different nominal wood-particle contents affect the mechanical response of PLA/wood material combinations before and after fungal colonisation. Furthermore, systematic comparisons between untreated and fungal-colonised specimens across several PLA/wood material combinations are still lacking. Consequently, the present study addresses an important and timely research gap in the field of bio-integrated additively manufactured materials.

Therefore, the present study investigates the tensile behaviour of additively manufactured PLA/wood biocomposites containing nominal wood-particle contents between 10 and 50 wt.%, both in the untreated condition and after post-printing colonisation with the fungus *Fomes fomentarius*. The underlying hypothesis of this work is that PLA/wood composites subjected to post-printing fungal colonisation and the associated processing conditions exhibit a modified mechanical response compared with untreated specimens, while providing a basis for future bio-integrated applications. Therefore, quantifying changes in tensile properties following fungal colonisation and the associated processing conditions represents an essential first step toward the development of functional hybrid bio-composite systems. The study focuses on experimentally comparing mean Young’s modulus (*E*), mean Ultimate Tensile Strength (UTS), and fracture behaviour under uniaxial tensile loading as a function of wood-particle content and post-printing fungal colonisation treatment. In addition, a supplementary screening-level Life Cycle Assessment (LCA) is conducted to compare the environmental impacts associated with the investigated material combinations during the MEX AM fabrication stage.

**Fig. 1:**
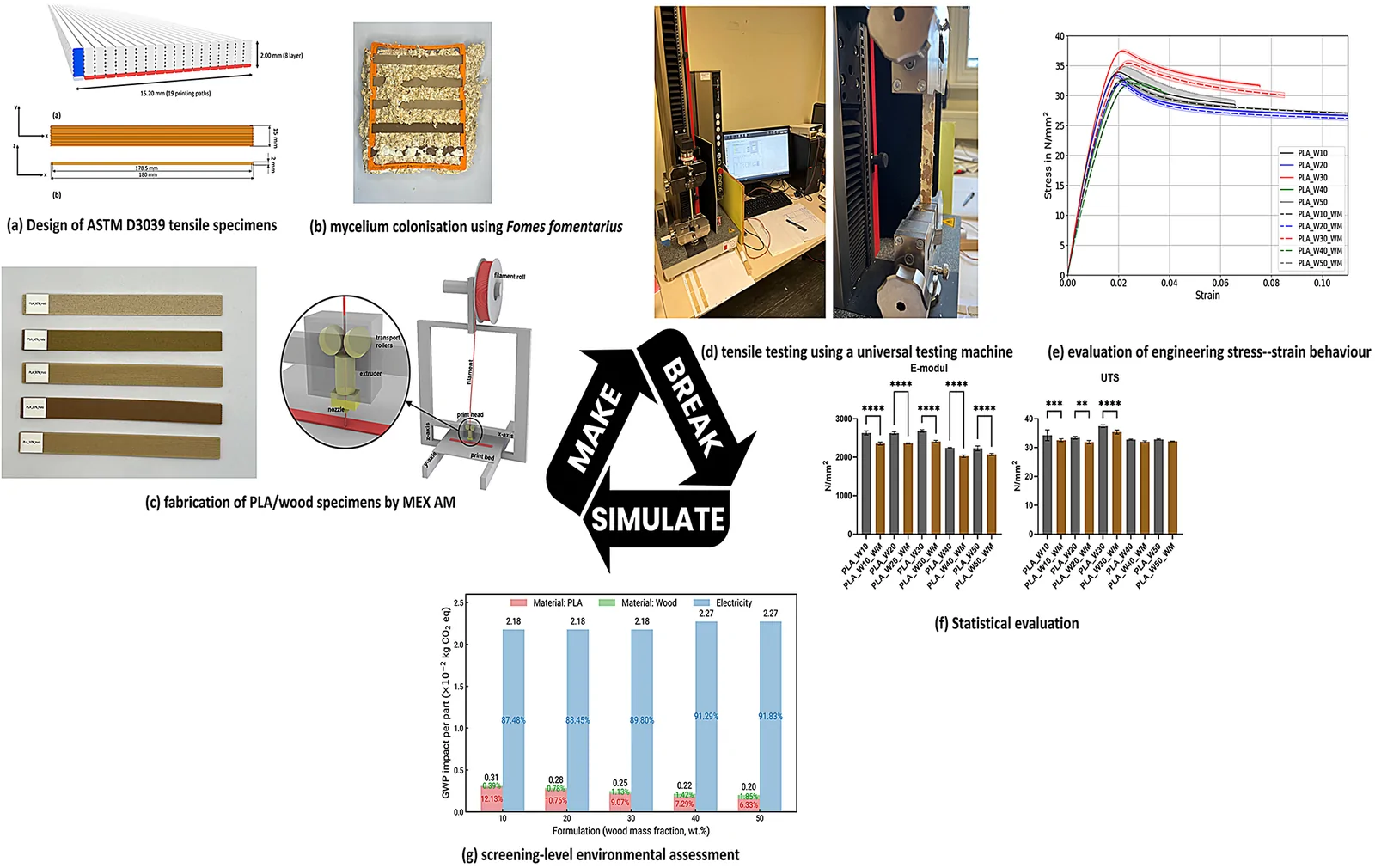
Overview of the Make–Break–Simulate workflow applied in this study: (**a**) design of the ASTM D3039 tensile specimen, (**b**) post-printing fungal colonisation using *Fomes fomentarius*, (**c**) fabrication of PLA/wood specimens using MEX AM, (**d**) tensile testing using a universal testing machine, (**e**) evaluation of engineering stress–strain behaviour and determination of mean *E* and mean UTS, (**f**) statistical analysis of untreated and fungal-colonised specimens, and (g) screening-level LCA of the investigated material combinations.

Figure 1 summarises the overall workflow of the study, including the fabrication of PLA/wood composite specimens by MEX AM, the subsequent fungal colonisation process, tensile characterisation, and the assessment of mechanical and environmental performance.

The following research questions are addressed in this work:

1.  How does the mechanical response of PLA/wood material combinations with different wood-particle contents differ between untreated specimens and specimens subjected to post-printing fungal colonisation and the associated processing conditions?
2. How do the investigated PLA/wood material combinations compare in terms of their screening-level environmental impacts during the MEX AM fabrication stage?

## Materials and methods

To investigate the tensile properties of PLA-based biocomposites with varying nominal wood-particle contents and subsequent fungal colonization, standardized tensile specimens were fabricated using MEX AM, also referred to as 3D-printing, and experimentally characterized under uniaxial tensile loading. The investigated material combinations consisted of commercially available PLA/wood composite filaments from different manufacturers, with nominal wood-particle contents ranging from 10 to 50 wt.%. Details of the individual filament products and manufacturers are provided in Section 2.1. In a second experimental series, selected specimens were subjected to post-printing colonization using the fungus *Fomes fomentarius* in order to compare the mechanical response of the printed material combinations before and after fungal colonization and the associated processing conditions.

The mechanical characterization focused on the evaluation of mean Young’s modulus (*E*) and mean ultimate tensile strength (UTS) obtained from the engineering stress–strain curves, while fracture surfaces were qualitatively examined using representative microscopy images. To ensure comparability between all investigated material combinations, all specimens were manufactured using identical MEX AM process parameters, specimen geometry, and tensile testing conditions. Tensile tests were performed using a universal testing machine in accordance with ASTM D3039. In addition to the mechanical investigation, a supplementary screening-level Life Cycle Assessment (LCA) was conducted to compare the environmental impacts associated with the investigated PLA/wood material combinations during the MEX AM fabrication stage.

### Make: raw filament materials

Commercially available PLA/wood composite filaments with nominal wood-particle contents of 10 wt.%^18^, 20 wt.%^19^, 30 wt.%^20^, 40 wt.%^21^, and 50 wt.%^22^ were investigated. The corresponding manufacturer technical data sheets are provided as Supplementary Material (Supplementary Material B). PLA is a bio-based thermoplastic derived from renewable feedstocks such as corn starch or sugar cane and is widely used in additive manufacturing due to its good printability and relatively high stiffness^23,24^. When combined with lignocellulosic fillers, e.g. wood particles, PLA can be processed into bio-based composite filaments suitable for MEX AM.

The investigated filaments were obtained as pre-compounded commercially available materials from different manufacturers and are summarised in Table 1.

**Table 1:** Material properties of the investigated PLA/wood composite filaments according to manufacturer specifications.

Property	PLA_W10	PLA_W20	PLA_W30	PLA_W40	PLA_W50
Material	ecoPLA Light	ecoPLA Dark	R3D Wood 1	EasyWood Pine	Wood Light Brown
Manufacturer	3DJAKE	3DJAKE	R3D	FormFutura	REDLINE FILAMENT
Nominal wood-particle content (wt.%)	10	20	30	40	50
Recommended nozzle temperature ($^\circ$C)	210–230	210–230	190–210	200–220	205 ± 10
Recommended bed temperature ($^\circ$C)	35–60	35–60	45–60	35–60	0–60
Manufacturer-specified UTS ($\mathrm {N/mm^2}$)	24.6	24.6	36.7	35	69.8
Manufacturer-specified strain at break (%)	23.8	23.8	6.0	5–10	4.8
Manufacturer-specified *E* ($\mathrm {N/mm^2}$)	370	370	3050	2000–3000	3120
Diameter (mm)	*1.75 ± 0.05*	*1.75 ± 0.05*	*1.75 ± 0.02*	*1.75 ± 0.05*	1.75

### Make: specimen fabrication

MEX AM was used to fabricate standardised tensile specimens for the mechanical investigation of the PLA/wood material combinations. MEX AM was selected due to its compatibility with commercially available PLA-based composite filaments and its widespread application in the processing of thermoplastic biocomposites^25–27^.

All investigated filament materials were processed using identical MEX AM process parameters to ensure comparability between the manufactured specimens. The selected process parameters were derived from preliminary printing trials and previous work reported in^14,28^. A nozzle diameter of 0.8 mm was used for all investigated material combinations.

**Fig. 2:**
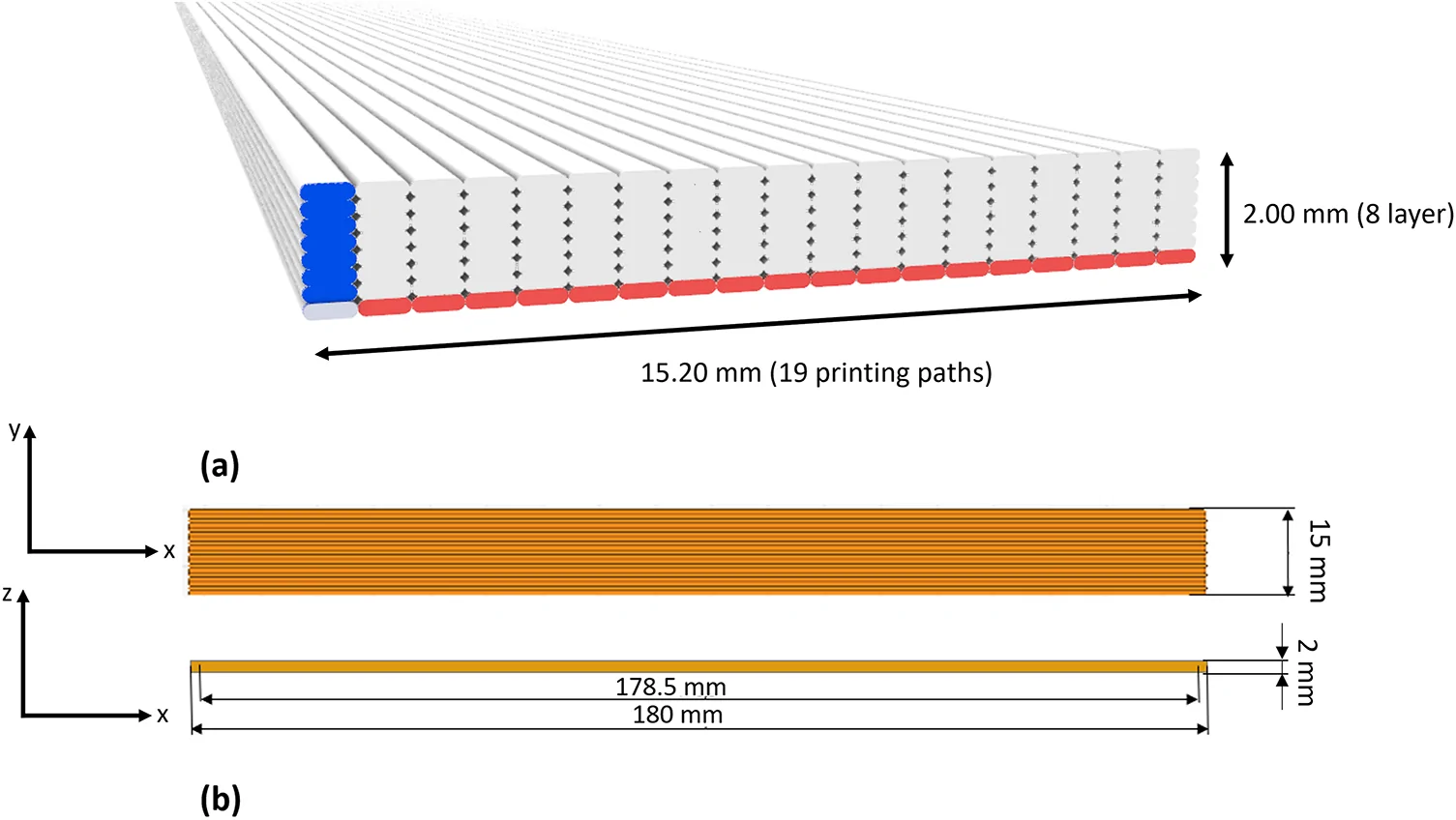
Overview of the additive manufacturing process and tensile specimen geometry used in this study: (**a**) schematic representation of the MEX AM process^28^ and (**b**) geometry of the tensile specimen according to ASTM D3039^28^.

Specimen fabrication was performed using a Prusa i3 MK3S+ (Prusa Research a.s., Prague, Czech Republic)^29^. In the applied MEX AM process, the filament material is transported into a heated extrusion system, melted, and deposited layer-wise through a nozzle onto the print bed. During printing, the print head moves along the *x*- and *z*-axes, while movement in the *y*-direction is realised by the print bed. A schematic representation of the MEX AM process is shown in Figure 2a.

The tensile specimens were designed according to ASTM D3039^30^. The specimen geometry was generated using Rhinoceros 3D (Rhino) (TLM, Inc., Seattle, USA)^31^. The final specimen geometry consisted of a total length of 180 mm, a free (gauge) length of $L_1 = 120$ mm, a clamping length of $L_2 = 30$ mm at each end, a width of *W = 15* mm, and a thickness of *H = 2* mm. The geometry of the tensile specimens is illustrated in Figure 2b.

**Fig. 3:**
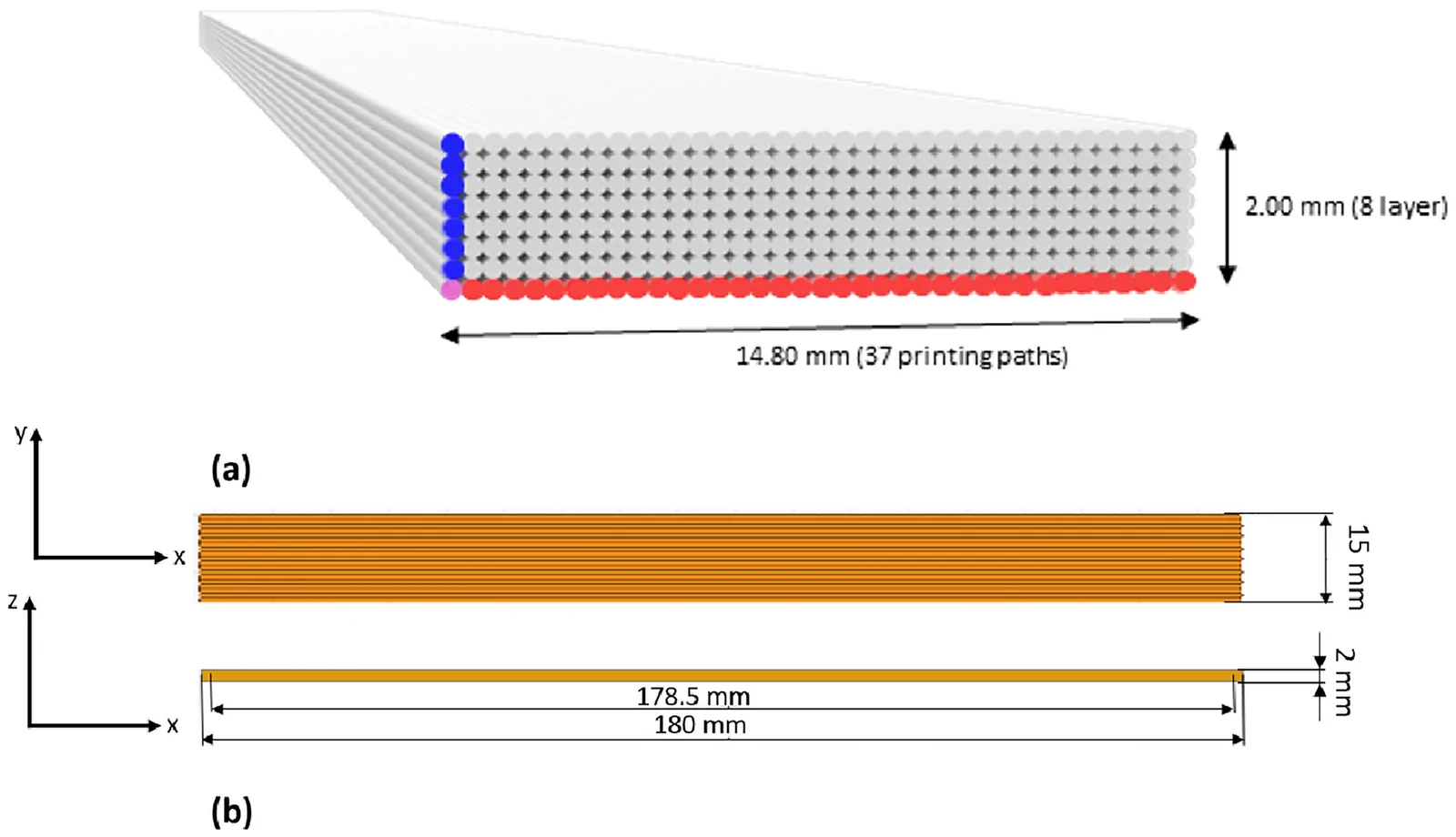
Tensile specimen geometry and raster configuration: (**a**) perspective view illustrating the deposited layers and extrusion paths. The red dots indicate the extrusion path of the first (bottom) layer, while the blue dots indicate the starting edge of each deposited layer and (**b**) raster orientation in plan and side view.

All specimens were manufactured using a 0$^\circ$ raster orientation, in which the deposited extrusion paths were aligned parallel to the loading direction. Raster orientation is known to significantly influence the tensile properties of MEX-manufactured thermoplastics and biocomposites. Previous studies have shown that extrusion paths aligned parallel to the loading direction generally result in improved tensile strength due to more efficient load transfer along continuous filament paths^32,33^. The resulting layer architecture and raster orientation applied during specimen fabrication are illustrated in Fig. 3.

Slicing and process parameter definition were performed using Ultimaker Cura (Version 5.2.1). An overview of the selected printing parameters is provided in Table 2. All specimens were manufactured using a layer height of 0.25 mm and a line width of 0.8 mm, resulting in eight deposited layers for the final specimen thickness of 2 mm. Each layer consisted of 19 parallel extrusion lines.

**Table 2:** Printing parameters according to Ultimaker Cura for the tensile specimens.

	**MEX AM Parameters**
Printer	Prusa i3 MK3S+
Nozzle Diameter	0.8 mm
	**Layer Parameters**
Layer Height	0.25 mm
Line Width	0.8 mm
Number of Layers	8
	**Raster Parameters**
Infill Density	100 %
Infill Pattern	Lines
Raster Orientation	0$^\circ$
	**Temperature Parameters**
Printing Temperature	190–205$^\circ$C
Bed Temperature	65$^\circ$C
	**Printing Parameters**
Print Speed	45 mm/s
Cooling	Off

To minimise moisture-related processing effects, all filament materials were stored under dry conditions prior to printing. PLA-based materials are known to exhibit moderate hygroscopic behaviour, and absorbed moisture can lead to hydrolytic degradation during thermal processing, resulting in unstable extrusion, pore formation, reduced interlayer bonding, and decreased mechanical performance^34–37^. Previous studies have further shown that moisture uptake during storage can reduce tensile strength and alter the mechanical response of additively manufactured PLA components, while appropriate drying procedures improve print quality and process stability^38–41^. Consequently, only newly opened and pre-dried filament materials were used throughout specimen fabrication to ensure reproducible manufacturing conditions.

For each investigated material combination, 16 tensile specimens were fabricated for mechanical testing, resulting in a total of 80 specimens across all investigated material combinations. Following specimen fabrication, half of the of the manufactured specimens (40) was subjected to post-printing fungal colonisation using *Fomes fomentarius*.

### Make: mycelium processing

**Fig. 4:**
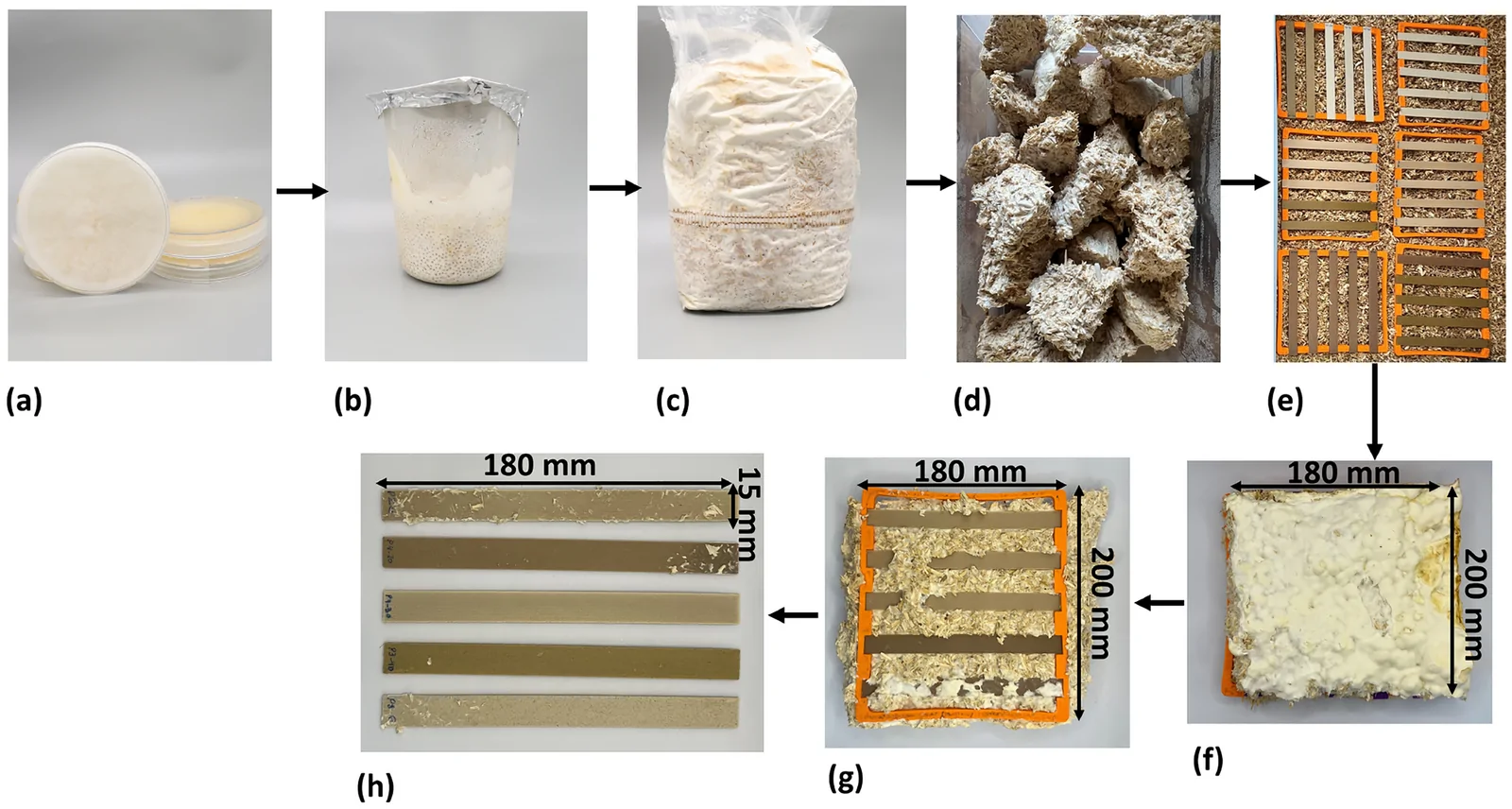
Overview of the mycelium processing and specimen colonisation procedure adapted from^14,43^: (**a**) agar plate pure culture of *Fomes fomentarius*, (**b**) colonised millet grain spawn, (**c**) hemp shive substrate after fungal colonisation, (**d**) shredded mycelium-colonised hemp substrate used as embedding material, (**e**) placement of additively manufactured PLA/wood tensile specimens on a bottom layer of shredded substrate within the cultivation container, (**f**) formation of a fully colonised mycelium–substrate–specimen block after colonisation, (**g**) extraction of the specimen carrier from the colonised block, and (**h**) removal of residual substrate and recovery of the individual tensile specimens prior to mechanical testing.

**Fig. 5:**
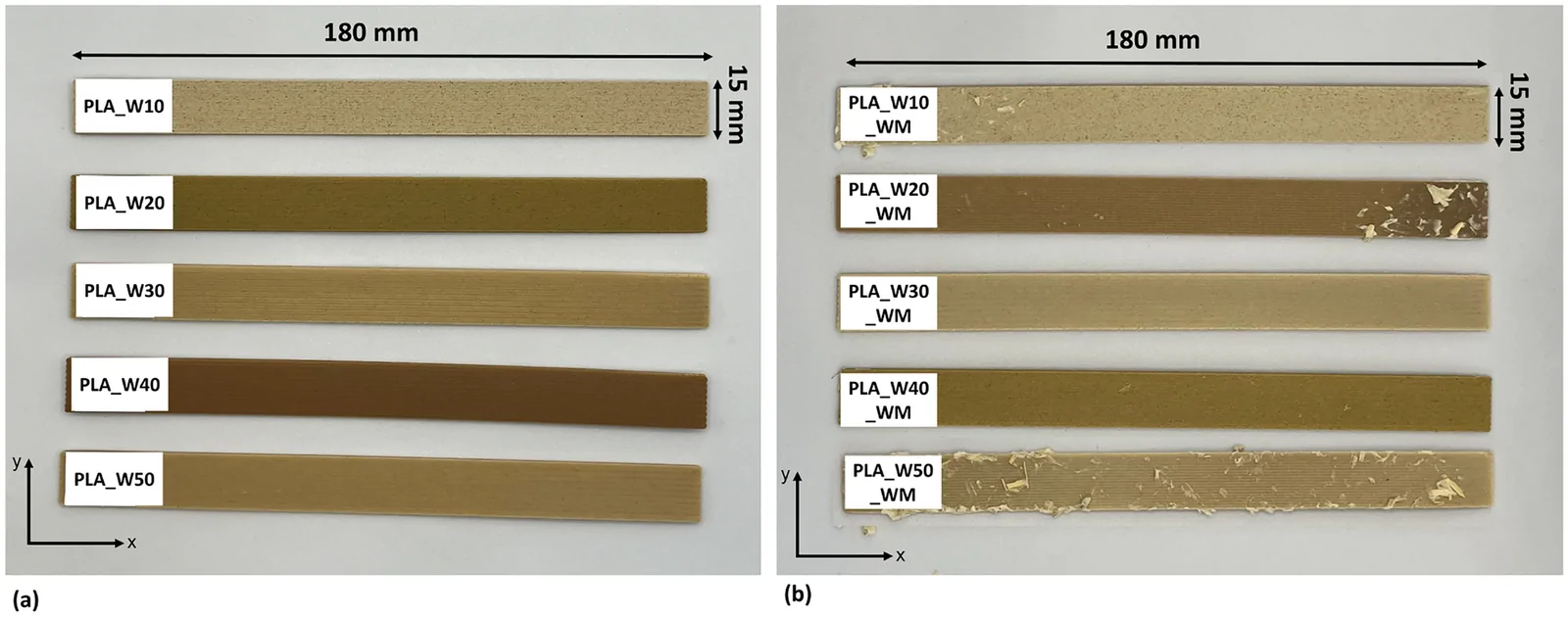
Overview of additively manufactured PLA/wood tensile specimens with different nominal wood-particle contents: (**a**) untreated specimens and (**b**) specimens after post-printing fungal colonisation using *Fomes fomentarius*.

Following MEX AM specimen fabrication, one half of the experimental specimens were prepared to investigate changes in the tensile properties of the additively manufactured PLA/wood material combinations following fungal colonisation and the associated processing conditions. For this purpose, 8 specimens from each of the five material combination were subjected to post-printing colonisation using the tinder fungus *Fomes fomentarius* (see Figure 4 and Figure 5).

The fungal strain *Fomes fomentarius* PaPF11 was cultivated under sterile laboratory conditions to minimise contamination by foreign microorganisms and ensure reproducible fungal growth. All cultivation procedures were performed under aseptic conditions using sterile workbenches and laminar flow cabinets. Materials and tools used during fungal cultivation were sterilised either by autoclaving or by treatment with 70 % ethanol. Colonisation was carried out in darkness at approximately 25,$^\circ$C following the cultivation procedure described by Pohl et al^42^. and Schmidt et al^43^., which is based on the methodology reported by Tacer-Caba et al^44^. The cultivation containers were maintained under humid conditions to support fungal growth. The relative humidity during the incubation period was not quantitatively monitored.

The cultivation procedure followed the methodology previously described in^14,43^ and is schematically illustrated in Figure 4. Initially, the fungal culture was propagated on agar plates (Figure 4a) and subsequently transferred onto sterilised millet grain spawn for approximately two weeks, resulting in complete mycelial overgrowth (Figure 4b). The colonised grain spawn was then used to inoculate hemp shives (Figure 4c), a lignocellulosic agricultural by-product selected due to its porous structure, lignin-rich composition, moisture retention capability, and suitability for homogeneous fungal colonisation^43^. During colonisation, the hemp substrate became uniformly colonised and interwoven by fungal hyphae.

Following colonisation, the mycelium-hemp substrate was shredded into smaller particles and used as embedding material for the printed tensile specimens. The shredded substrate was first distributed as a bottom layer inside the cultivation containers, after which the specimens were positioned using specimen holders to ensure reproducible spacing and orientation (Figure 4d). Subsequently, an upper layer of colonised substrate was added to fully surround the specimens. Gentle manual compaction was applied to ensure continuous contact between the substrate and specimen surfaces and to minimise hollow spaces. No quantitative compaction pressure was applied or recorded.

The assemblies were then incubated for an additional period of two weeks, during which the fungal mycelium overgrew the shredded substrate and surrounding specimen surfaces, resulting in cohesive mycelium-substrate-specimen blocks (Fig. 4e–f)^14^.

After completion of the colonisation process, the colonised assemblies were dried at 55,$^\circ$C in order to reduce residual moisture and terminate biological activity^45–47^. Subsequently, the specimens were extracted from the surrounding substrate, residual mycelium was carefully removed from the specimen surfaces, and the samples were vacuum sealed prior to mechanical testing^14,43^.

### Break: experimental setup and testing procedure

Mechanical characterisation of the additively manufactured tensile specimens was performed using a Universal Testing Machine (UTM), model ZwickRoell Z2.5 (ZwickRoell GmbH & Co. KG, Ulm, Germany). The testing machine provides a maximum load capacity of 2.5 kN and allows precise control of displacement and crosshead speed, making it suitable for tensile testing of PLA-based composite materials.

The tensile tests were conducted in accordance with ASTM D3039^30^. During testing, the specimens were mounted using pneumatic grips to minimise slippage and ensure reproducible load transfer throughout the experiments. All tests were performed under displacement-controlled loading conditions until specimen failure occurred.

A constant crosshead displacement rate of 2 mm/min was applied for all tensile tests. This loading rate was selected to ensure quasi-static loading conditions and to allow direct comparison with previous investigations on additively manufactured polymer and bio-composite structures^14^. Similar displacement rates have also been widely employed in tensile characterisation studies of MEX-manufactured PLA-based composites and natural-fibre-reinforced polymer systems, where low crosshead speeds are recommended to minimise rate-dependent effects and to ensure stable acquisition of the elastic response^9–11^.

Throughout the experiments, force and displacement data were continuously recorded by the testing system. Engineering stress–strain curves were subsequently calculated from the recorded raw data, with engineering strain derived from the recorded crosshead displacement relative to the initial gauge length. Mean *E* and mean UTS were determined from the resulting displacement-derived engineering stress–strain curves in accordance with ASTM D3039^30^. Young’s modulus was calculated from the initial linear elastic region of the displacement-derived stress–strain response, whereas the UTS was defined as the maximum engineering stress recorded during the tensile test. These parameters were selected because they represent the primary descriptors of tensile stiffness and load-bearing capacity for fibre- and particle-reinforced composite materials subjected to uniaxial loading^30,48^.

Statistical evaluation was performed separately for each investigated material combination by comparing untreated specimens with the corresponding fungal-colonised specimens possessing the same nominal wood-particle content. The resulting values of mean *E* and mean UTS were subsequently used for statistical analysis, as described in Section 2.5.

**Fig. 6:**
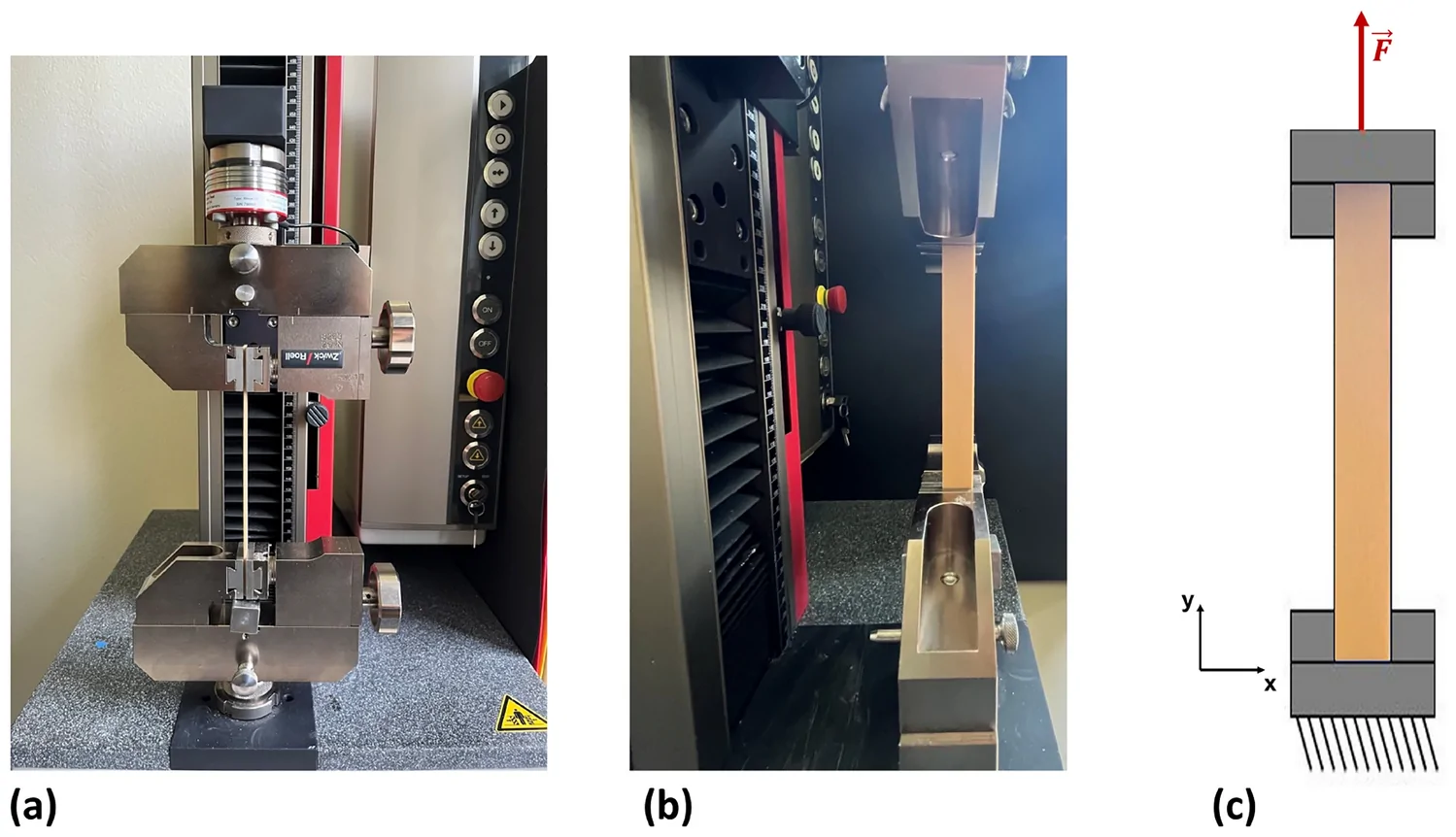
Tensile testing configuration used in this study: (**a**) front view of the experimental setup using the ZwickRoell Z2.5 universal testing machine, (**b**) side view of a mounted PLA/wood tensile specimen during testing, and (**c**) schematic representation of the uniaxial tensile loading configuration indicating the loading direction and specimen orientation.

Figure 6 illustrates the experimental tensile testing setup and the schematic loading configuration used in this study. Visual inspection of the fractured specimens confirmed that failure occurred within the gauge section rather than in the clamping regions. Most specimens fractured approximately in the central region of the specimen, although the exact fracture location varied between individual specimens.

### Break: statistical analysis

Statistical analyses were performed to evaluate differences in mean *E* and mean UTS between untreated PLA/wood material combinations and specimens subjected to fungal colonisation. For each material combination, eight specimens were tested, and the resulting values of mean *E* and mean UTS were used for statistical evaluation.

All statistical analyses were conducted using GraphPad Prism (Version 10.6.1)^49^. Mean values, standard deviations (SD), and 95 % confidence intervals were calculated for each specimen group. One-way ANOVA was performed separately for each commercial PLA/wood material combination. A two-way ANOVA was intentionally not applied because the investigated commercial filaments originated from different manufacturers and may differ not only in nominal wood-particle content but also in PLA grade, wood species, particle morphology, additives, and compounding procedures. Consequently, the investigated material combinations cannot be considered as levels of a single independent experimental factor.

To assess differences in the mean *E* and mean UTS of each material combination between untreated and fungal-colonised specimens, one-way Analysis Of Variance (ANOVA) followed by Tukey’s post-hoc test was performed^50^. Statistical significance was evaluated using the conventional significance thresholds of *p<0.05, **p<0.01, ***p<0.001, and ****p<0.0001.

The *p*-value represents the probability of observing a difference at least as large as that measured if no true difference exists between the compared groups (null hypothesis). Consequently, lower *p*-values indicate stronger statistical evidence against the null hypothesis. Differences were considered statistically significant when *p<0.05*, whereas results with *p\ge 0.05* were considered not statistically significant (ns).

The statistical evaluation focused on pairwise comparisons between untreated PLA/wood material combinations and the corresponding fungal-colonised specimens (WM) for each investigated wood-particle content.

### Simulate: life cycle assessment methodology

A supplementary screening-level Life Cycle Assessment (LCA) is conducted to compare the environmental impacts associated with the investigated PLA/wood material combinations during the printing stage of MEX AM. The assessment is structured with reference to the goal-and-scope, inventory, impact assessment, and interpretation framework of ISO 14040 and ISO 14044^51,52^, but it is not intended as a full ISO-compliant product LCA. The assessment builds on a previously published explainable AI-assisted environmental assessment (XAI-LCA) workflow^53^. The procedure specific to the present study is summarised below.

The assessment supports the comparative interpretation of the indicative environmental performance of the five investigated PLA/wood material combinations within the defined printing-stage system boundary. The foreground system is limited to MEX AM specimen fabrication, including electricity use during printing and material-combination-dependent material consumption. Upstream impacts associated with the consumed electricity and raw-material production are included through background datasets and literature-based characterization factors. The post-printing fungal colonisation process, including substrate preparation, incubation, drying, specimen extraction, and storage, is outside the system boundary of the present screening assessment. The use phase and end-of-life stage are also excluded. Three midpoint indicators are considered: Global Warming Potential (GWP), Acidification Potential (AP), and Eutrophication Potential (EP).

The declared unit is one printed tensile specimen manufactured under the defined MEX AM conditions, corresponding to one individual print job as described in the previous sections. For each material combination, the printing time is obtained from the slicing software and used to calculate the electricity consumption $E_{\textrm{el}}$ (kWh) through the calibrated average-power approach described in the previously published XAI–LCA workflow^53^. The calculated electricity consumption is modelled as an input to the foreground printing process in openLCA (v2.3.1)^54^. The electricity-related midpoint impacts are calculated using a German low-voltage electricity background dataset from the ELCD data package and the TRACI 2.1 midpoint method^55^.

The total specimen mass $m_{\textrm{tot}}$ (kg) is determined using a precision digital balance. The nominal wood-particle content *w* (dimensionless, 0–1 by mass) is used to split the total specimen mass into PLA and wood contributions:

1 $$\begin{aligned} m_{\textrm{PLA}} = (1-w) \cdot m_{\textrm{tot}}, \qquad m_{\textrm{wood}} = w \cdot m_{\textrm{tot}}, \end{aligned}$$

where $m_{\textrm{wood}}$ is the wood-particle mass, $m_{\textrm{PLA}}$ is the PLA mass, $m_{\textrm{tot}}$ is the measured total specimen mass, and *w* is the nominal wood-particle content expressed as a mass fraction.

Because suitable polymer and wood production datasets are not consistently available within the selected database, material-related impacts are calculated using literature-based cradle-to-gate characterization factors (Table 3). These factors are linearly scaled with the measured material masses:

2 $$\begin{aligned} I^{\textrm{mat}}_k = m_{\textrm{PLA}} \cdot \textrm{CF}_{k,\textrm{PLA}} + m_{\textrm{wood}} \cdot \textrm{CF}_{k,\textrm{wood}}, \end{aligned}$$

where $I^{\textrm{mat}}_k$ is the material-related impact for midpoint indicator *k*, $\textrm{CF}_{k,\textrm{PLA}}$ is the characterization factor for PLA, and $\textrm{CF}_{k,\textrm{wood}}$ is the characterization factor for wood.

**Table 3:** Cradle-to-gate characterization factors used to map material consumption to midpoint indicators.

Material	Indicator	Unit (per kg)	Factor	Reference
PLA resin	GWP	kg CO$_2$ eq/kg	0.502	^56^
PLA resin	AP	kg SO$_2$ eq/kg	0.021	^56^
PLA resin	EP	kg N eq/kg	0.0133	^57^
Wood flour	GWP	kg CO$_2$ eq/kg	0.14633	^58^
Wood flour	AP	kg SO$_2$ eq/kg	0.00147	^58^
Wood flour	EP	kg N eq/kg	$7.63\times 10^{-5}$	^58^

It should be noted that the electricity- and material-related impact contributions are derived from different secondary data sources. The electricity-related impacts are calculated in openLCA using a German low-voltage electricity background dataset and the TRACI 2.1 midpoint method, whereas the PLA- and wood-related impact contributions are estimated using literature-based cradle-to-gate characterization factors. Although consistent units are used for each midpoint indicator, the underlying datasets may differ in terms of system boundaries, geographical and temporal representativeness, background modelling assumptions, and LCIA characterization basis. Therefore, the calculated values are intended to support the comparative interpretation of indicative trends among the investigated PLA/wood material combinations, rather than precise comparisons of absolute environmental burdens.

For each midpoint indicator *k*, the total printing-stage impact is obtained by combining electricity- and material-related contributions:

3 $$\begin{aligned} I^{\textrm{tot}}_k = I^{\textrm{elec}}_k + I^{\textrm{mat}}_k, \end{aligned}$$

where $I^{\textrm{tot}}_k$ is the total printing-stage impact, $I^{\textrm{elec}}_k$ is the electricity-related impact, and $I^{\textrm{mat}}_k$ is the material-related impact for midpoint indicator *k*.

The resulting midpoint impacts are used to compare indicative environmental trends among the different PLA/wood material combinations and to support the subsequent interpretation of the trade-off between mechanical performance and printing-stage environmental burden under the assumptions of the present screening assessment.

## Results

The mechanical behaviour of the investigated PLA/wood material combinations was evaluated by uniaxial tensile testing. The analysis focused on differences in the engineering stress–strain response, mean *E*, and mean UTS between the investigated material combinations and between untreated and fungal-colonised specimens.

For all investigated material combinations, mean values, standard deviations, and 95 % confidence intervals were calculated based on eight specimens per test series.

### Stress–strain behaviour

**Fig. 7:**
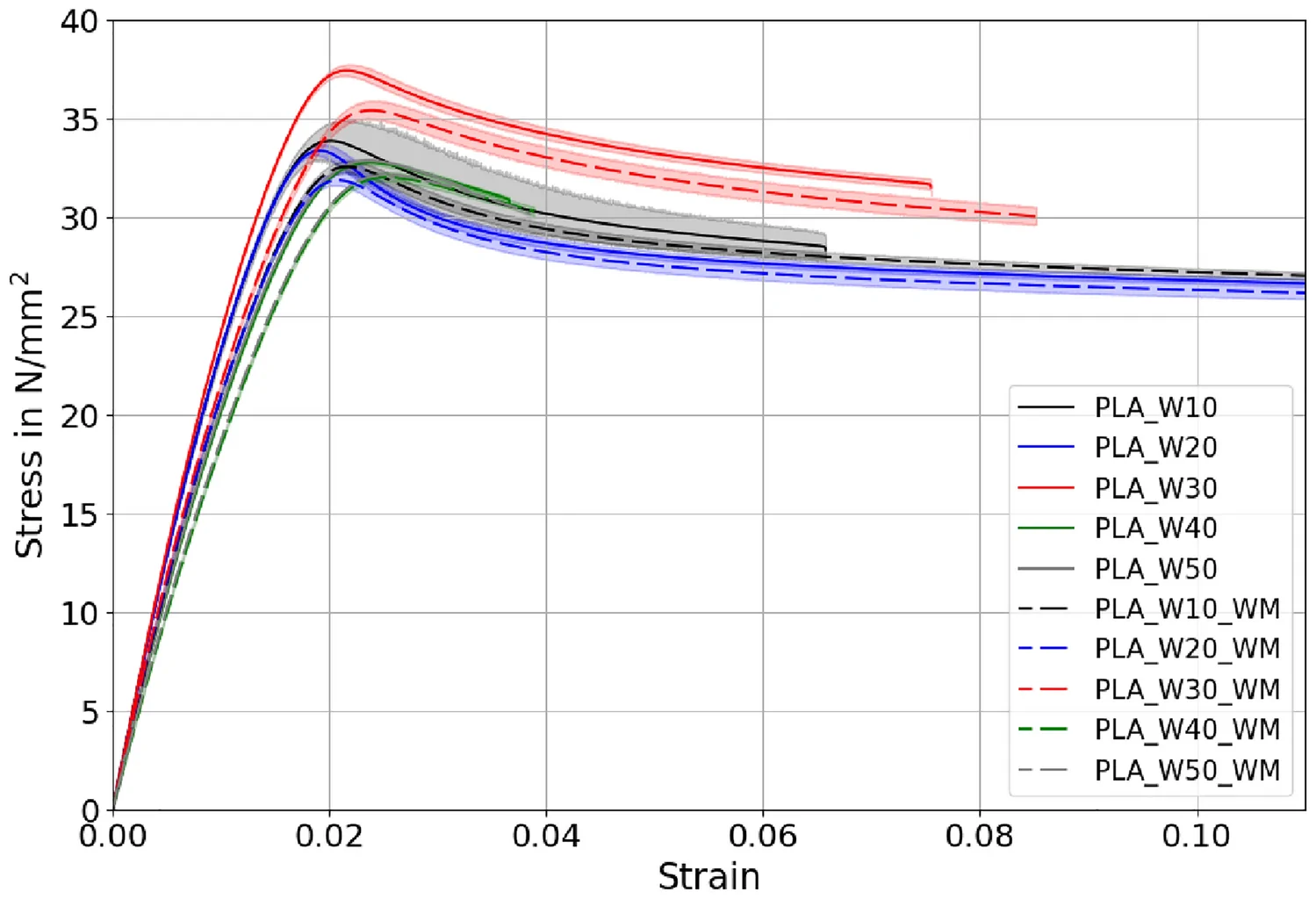
Mean engineering stress–strain curves of all investigated PLA/wood material combinations. Solid lines represent untreated specimens, whereas dashed lines indicate fungal-colonised specimens (WM). Colours correspond to the nominal wood-particle content (10–50 wt.%). Shaded regions denote the corresponding 95 % confidence intervals.

Figure 7 presents the mean engineering stress–strain curves for all investigated material combinations. Solid lines represent untreated PLA/wood material combinations, whereas dashed lines correspond to specimens subjected to fungal colonisation (WM). The coloured curves indicate the different nominal wood-particle contents: black (PLA_W10), blue (PLA_W20), red (PLA_W30), green (PLA_W40), and grey (PLA_W50). The shaded regions represent the corresponding 95 % confidence intervals.

The stress–strain curves reveal clear differences between the investigated material combinations. For all material combinations, an initial approximately linear elastic region is followed by a maximum stress level corresponding to the mean UTS, after which a gradual reduction in stress is observed until specimen failure. Both the initial slope of the curves and the maximum stress values varied depending on the wood-particle content and specimen treatment condition.

Mean *E* was calculated from the initial linear region of the engineering stress–strain curves in accordance with ASTM D3039^30^ and mean UTS was determined as the maximum engineering stress recorded during the tensile test. The resulting mean *E* and mean UTS are summarised in Table 4. All values of mean *E* and mean UTS are reported in $\mathrm {N/mm^2}$.

A visual comparison of the stress–strain curves indicates noticeable differences between the investigated material combinations. Among the tested commercial filaments, the PLA_W30 material combination exhibited the highest measured mean UTS.

**Table 4:** Mean Young’s modulus (*E*) and mean Ultimate Tensile Strength (UTS) of additively manufactured PLA/wood material combinations with and without fungal colonisation. Values are reported as mean ± standard deviation (SD).  Fungal-colonised specimens underwent a two-week colonisation period followed by drying and specimen recovery prior to mechanical testing.

Specimen	*E* ($\mathrm {N/mm^2}$)		UTS ($\mathrm {N/mm^2}$)		Colonisation
	Mean	SD	Mean	SD	Duration
PLA_W10	2631.74	18.21	34.29	0.66	–
PLA_W20	2629.49	11.77	33.62	0.16	–
PLA_W30	2685.01	10.23	37.98	0.16	–
PLA_W40	2242.16	5.04	32.78	0.07	–
PLA_W50	2231.05	20.33	32.86	0.07	–
PLA_W10_WM	2355.21	12.65	32.55	0.17	2 weeks
PLA_W20_WM	2360.39	4.50	31.88	0.20	2 weeks
PLA_W30_WM	2409.32	9.70	35.41	0.25	2 weeks
PLA_W40_WM	2025.39	9.14	32.01	0.12	2 weeks
PLA_W50_WM	2072.72	8.80	32.14	0.05	2 weeks

#### PLA/wood material combinations with 10 wt.% wood-particle content

**Fig. 8:**
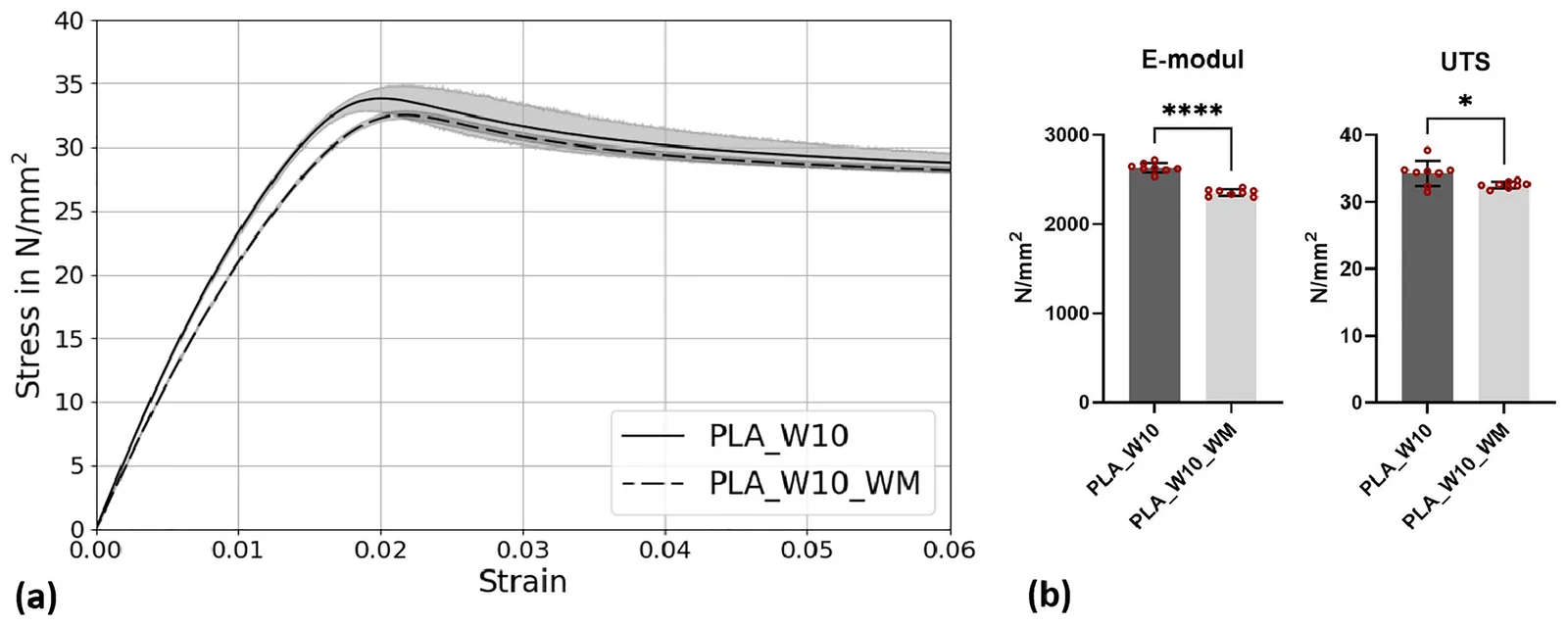
Mechanical characterisation of PLA_W10 and PLA_W10_WM specimens: (a) mean engineering stress–strain curves with corresponding 95 % confidence intervals and (b) comparison of mean *E* and mean UTS. Error bars represent standard deviations. Statistical significance was evaluated using one-way ANOVA followed by Tukey’s post-hoc test (*p<0.05, **p<0.01, ***p<0.001, ****p<0.0001).

Figure 8 summarises the mechanical response of both material combinations. Figure 8a presents the mean engineering stress–strain curves together with their 95 % confidence intervals, whereas Fig. 8b compares the resulting values of mean *E* and mean UTS. Both material combinations exhibited a similar overall tensile response, characterised by an initial approximately linear elastic region followed by a maximum stress level corresponding to the mean UTS. Compared with the untreated specimens, the fungal-colonised specimens exhibited a lower initial slope of the stress–strain curve and lower maximum stress values, consistent with the lower mean *E* and mean UTS measured for the fungal-colonised group.

**Fig. 9:**
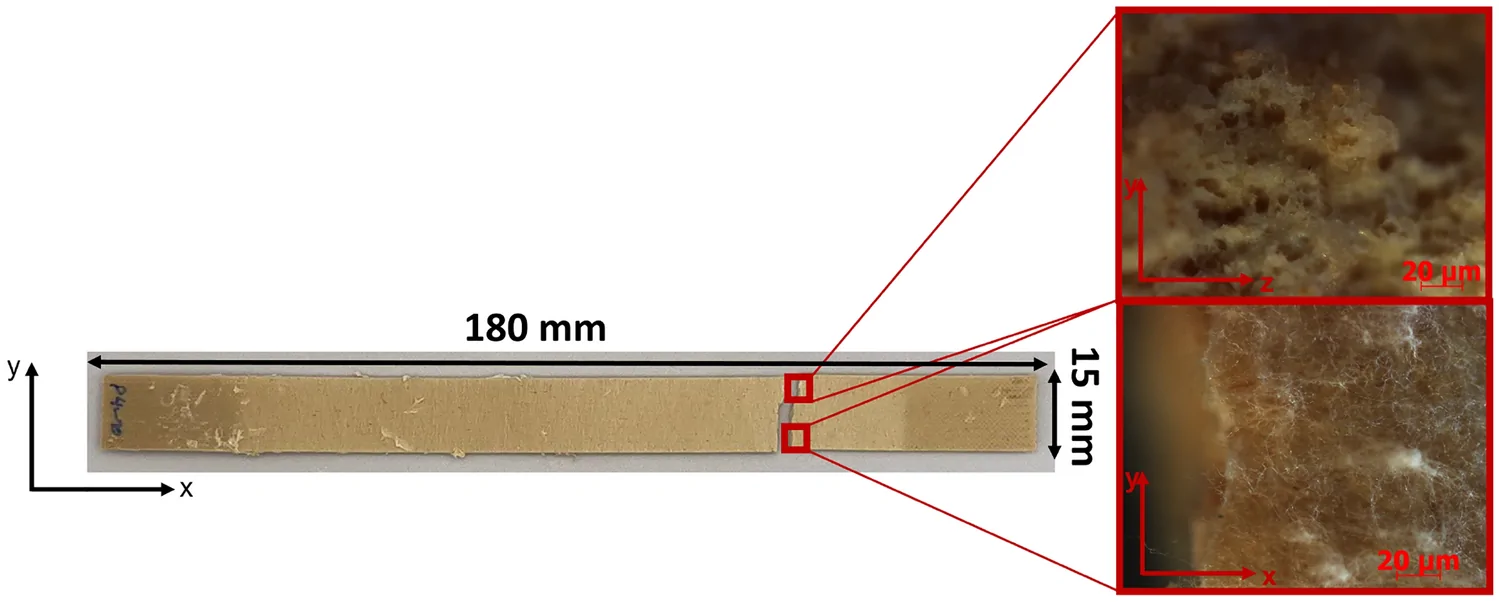
Macroscopic overview and representative microscopy images of a fungal-colonised PLA_W10_WM specimen after two weeks of colonisation with *Fomes fomentarius*. The highlighted regions indicate locations selected for detailed examination. The upper micrograph shows the specimen cross-section (*y*–*z* plane), whereas the lower micrograph presents the specimen surface (*x*–*y* plane). Fungal structures are visible at the analysed locations. Scale bars correspond to 20 *μ*m.

Representative macroscopic and microscopic observations of fungal-colonised specimens are shown in Fig. 9. The highlighted regions were selected for microscopic examination after the two-week colonisation period by *Fomes fomentarius*. The microscopic observations reveal the presence of fungal structures on the specimen surface, confirming successful surface colonisation of the PLA/Wood specimens by *Fomes fomentarius*. However, the present observations do not allow conclusions regarding the extent of fungal growth within the internal specimen structure.

The untreated PLA/Wood composite containing 10 wt.% wood-particle content (PLA_W10) exhibited a mean *E* of $2631.74 \pm 18.21~\mathrm {N/mm^2}$ and a mean UTS of $34.29 \pm 0.66~\mathrm {N/mm^2}$. Following fungal colonisation (PLA_W10_WM), mean *E* decreased to $2355.21 \pm 12.65~\mathrm {N/mm^2}$ and the mean UTS decreased to $32.55 \pm 0.17~\mathrm {N/mm^2}$. These changes correspond to reductions of 10.5 % for mean *E* and 5.1 % for the mean UTS.

A one-way ANOVA followed by Tukey’s post-hoc test revealed statistically significant differences between PLA_W10 and PLA_W10_WM for both mean *E* (****p < 0.0001) and mean UTS (*p < 0.05).

Overall, the PLA_W10 material combination exhibited statistically significant differences in both mean *E* and mean UTS between untreated and fungal-colonised specimens. The observed differences between PLA_W10 and PLA_W10_WM demonstrate that even at the lowest investigated wood-particle content,  the fungal-colonised specimen group exhibited a measurably different tensile response compared with the untreated reference group. These findings are consistent with the general trend observed across all investigated PLA/wood material combinations.

#### PLA/Wood material combinations with 20 wt.% wood-particle content

**Fig. 10:**
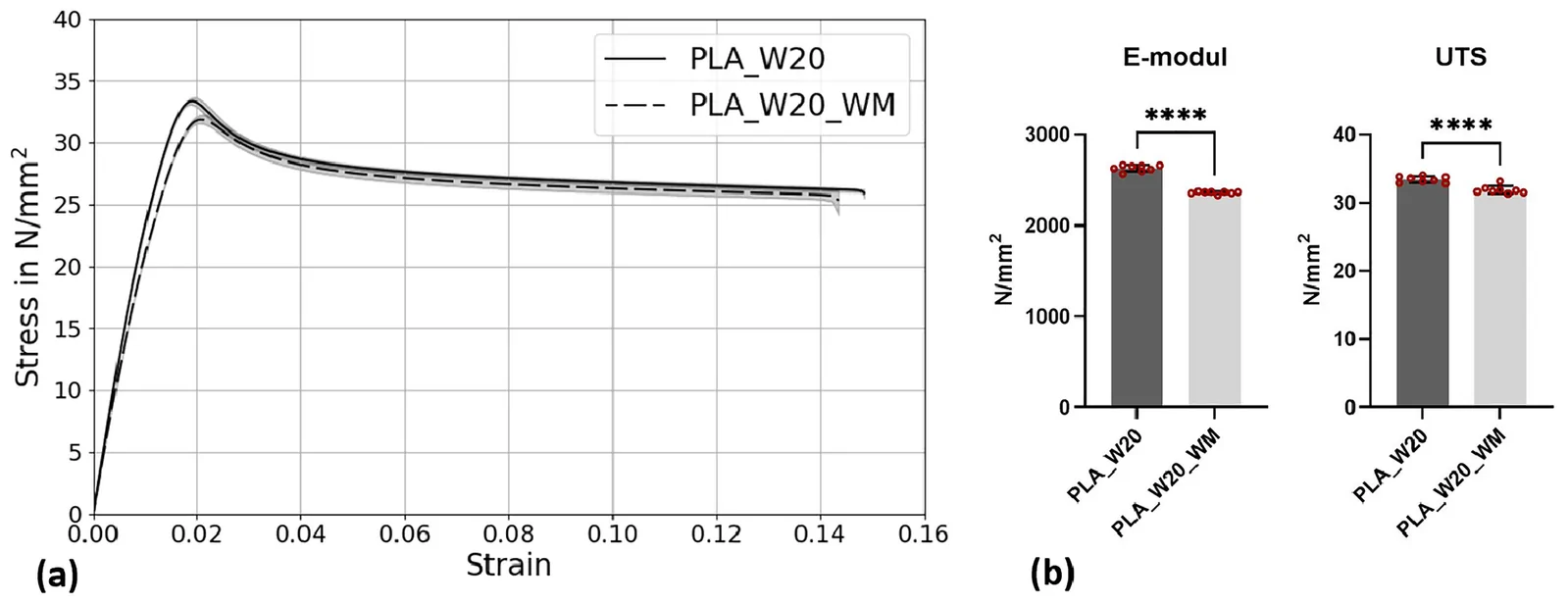
Mechanical characterisation of PLA_W20 and PLA_W20_WM specimens: (a) mean engineering stress–strain curves with corresponding 95 % confidence intervals and (b) comparison of mean *E* and mean UTS. Error bars represent standard deviations. Statistical significance was evaluated using one-way ANOVA followed by Tukey’s post-hoc test (*p<0.05, **p<0.01, ***p<0.001, ****p<0.0001).

Figure 10 summarises the mechanical response of both material combinations. Figure 10a presents the mean engineering stress–strain curves together with their 95 % confidence intervals, whereas Fig. 10b compares the resulting values of mean *E* and mean UTS. Both material combinations exhibited a similar overall tensile response, characterised by an initial approximately linear elastic region followed by a pronounced stress peak at a strain of approximately 0.02, corresponding to the mean UTS. Following this peak, both material combinations exhibited a noticeable reduction in stress before reaching a more gradual post-peak region. Compared with the untreated specimens, the fungal-colonised specimens exhibited a lower initial slope of the stress–strain curve and lower maximum stress values, which is consistent with the reductions observed for both mean *E* and mean UTS.

**Fig. 11:**
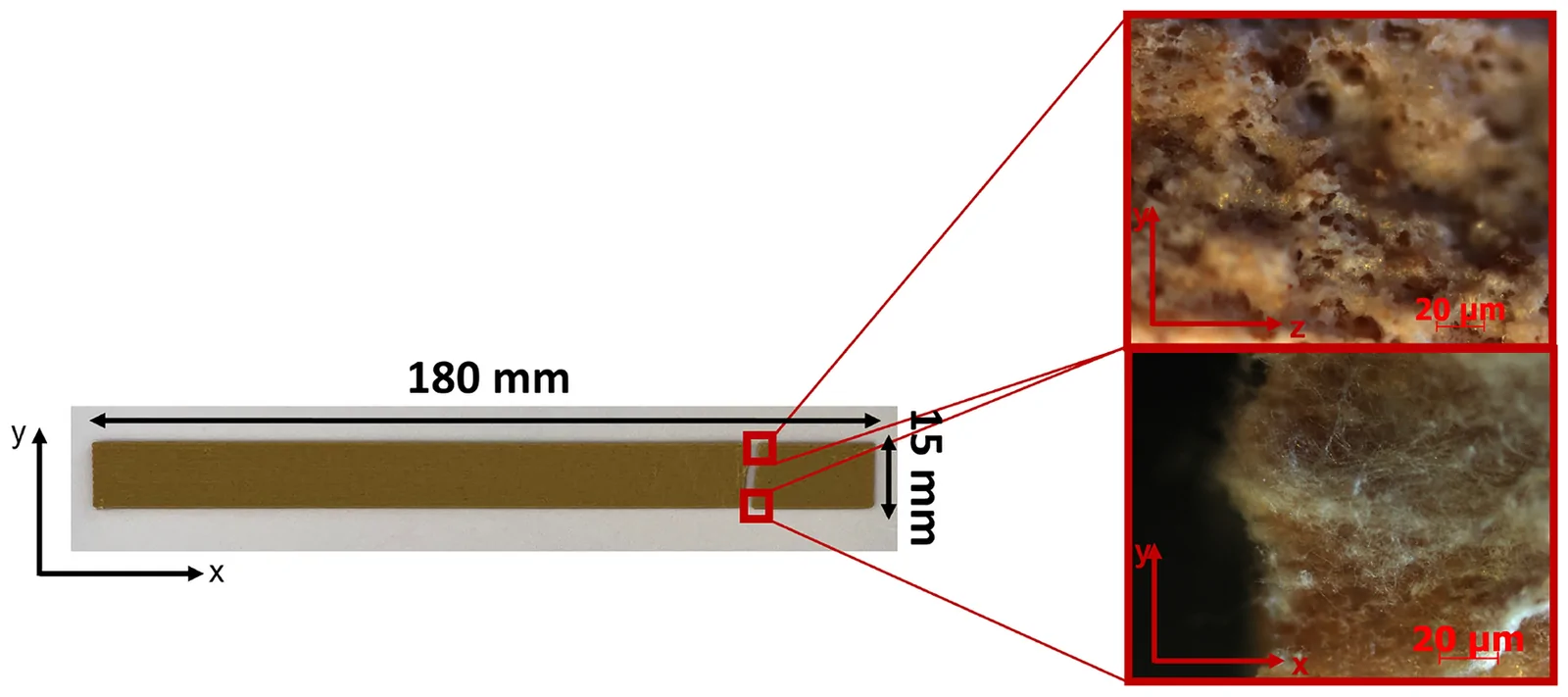
Macroscopic specimen view and microscopy analysis of PLA_W20_WM after fungal colonisation by *Fomes fomentarius*. Selected regions of interest are indicated in the overview image. Representative microscopic observations obtained from the cross-sectional area (*y*–*z* plane) and the outer specimen surface (*x*–*y* plane) confirm the presence of fungal structures after colonisation. Scale bars correspond to 20 *μ*m.

Representative macroscopic and microscopic observations of fungal-colonised specimens are shown in Fig. 11. The highlighted regions were selected for microscopic examination after the two-week colonisation period. The microscopic observations reveal the presence of fungal structures on the specimen surface, confirming successful surface colonisation of the PLA/Wood specimens by *Fomes fomentarius*. Similar to the observations for PLA_W10, fungal structures were primarily observed at the specimen surface. However, the present observations do not allow conclusions regarding the extent of fungal growth within the internal specimen structure.

For the material combination containing 20 wt.% wood-particle content (PLA_W20), the measured mean *E* was $2629.49 \pm 11.77~\mathrm {N/mm^2}$ and the mean UTS reached $33.62 \pm 0.16~\mathrm {N/mm^2}$. Following fungal colonisation (PLA_W20_WM), both properties decreased to lower values, corresponding to reductions of 10.2 % in mean *E* and 5.2 % in mean UTS.

A one-way ANOVA followed by Tukey’s post-hoc test revealed statistically significant differences between PLA_W20 and PLA_W20_WM for both mean *E* (****p < 0.0001) and mean UTS (**p < 0.01).

In summary, the fungal-colonised specimens exhibited measurable reductions in both investigated mean *E* and mean UTS relative to the untreated specimens. The magnitude of these changes was comparable to that observed for PLA_W10, indicating a similar response of the material to the applied colonisation and associated processing conditions.

#### PLA/wood material combinations with 30 wt.% wood-particle content

**Fig. 12:**
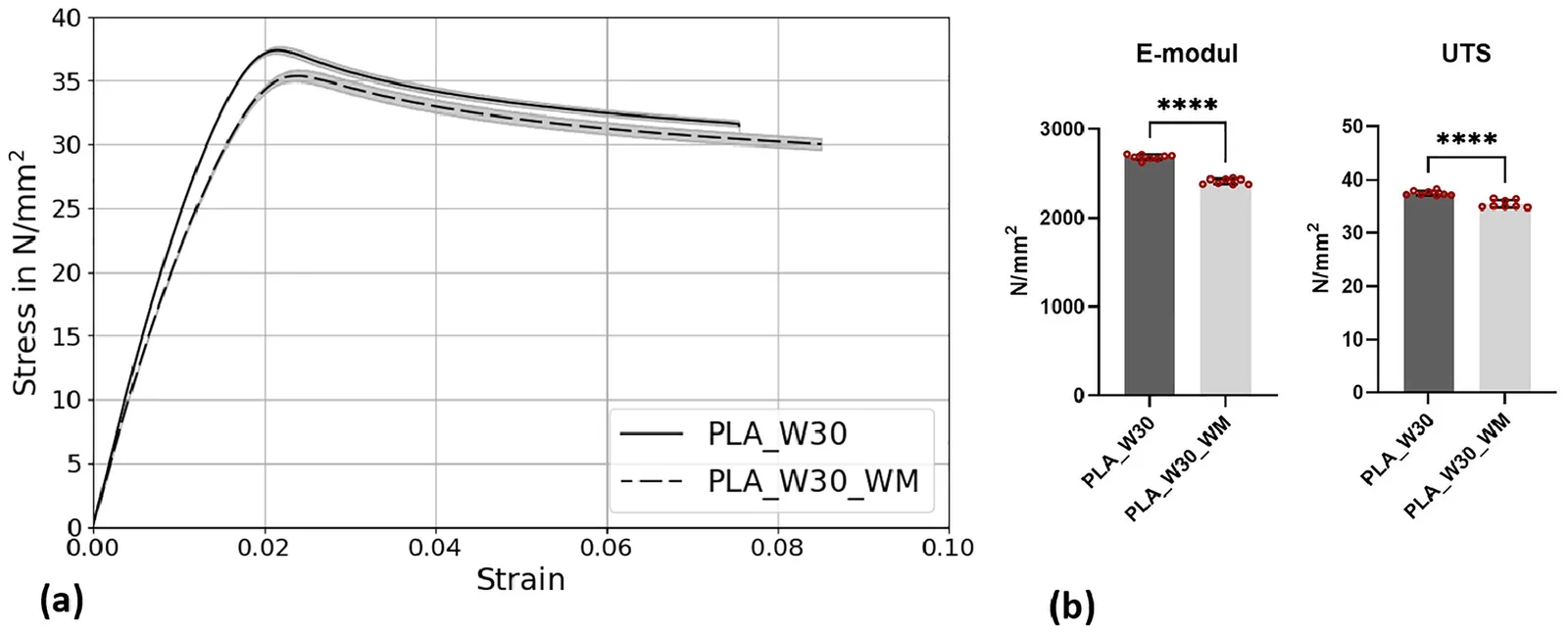
Mechanical characterisation of PLA_W30 and PLA_W30_WM specimens: (a) mean engineering stress–strain curves with corresponding 95 % confidence intervals and (b) comparison of mean *E* and mean UTS. Error bars represent standard deviations. Statistical significance was evaluated using one-way ANOVA followed by Tukey’s post-hoc test (*p<0.05, **p<0.01, ***p<0.001, ****p<0.0001).

Figure 12 summarises the mechanical response of both material combinations. Figure 12a presents the mean engineering stress–strain curves together with their 95 % confidence intervals, whereas Fig. 12b compares the resulting values of mean *E* and mean UTS. Both material combinations exhibited a similar overall tensile response, characterised by an initial approximately linear elastic region followed by a maximum stress level corresponding to the mean UTS. Compared with the untreated specimens, the fungal-colonised specimens exhibited a lower initial slope of the stress–strain curve and lower maximum stress values, which is consistent with the reductions observed for both mean *E* and mean UTS.

**Fig. 13:**
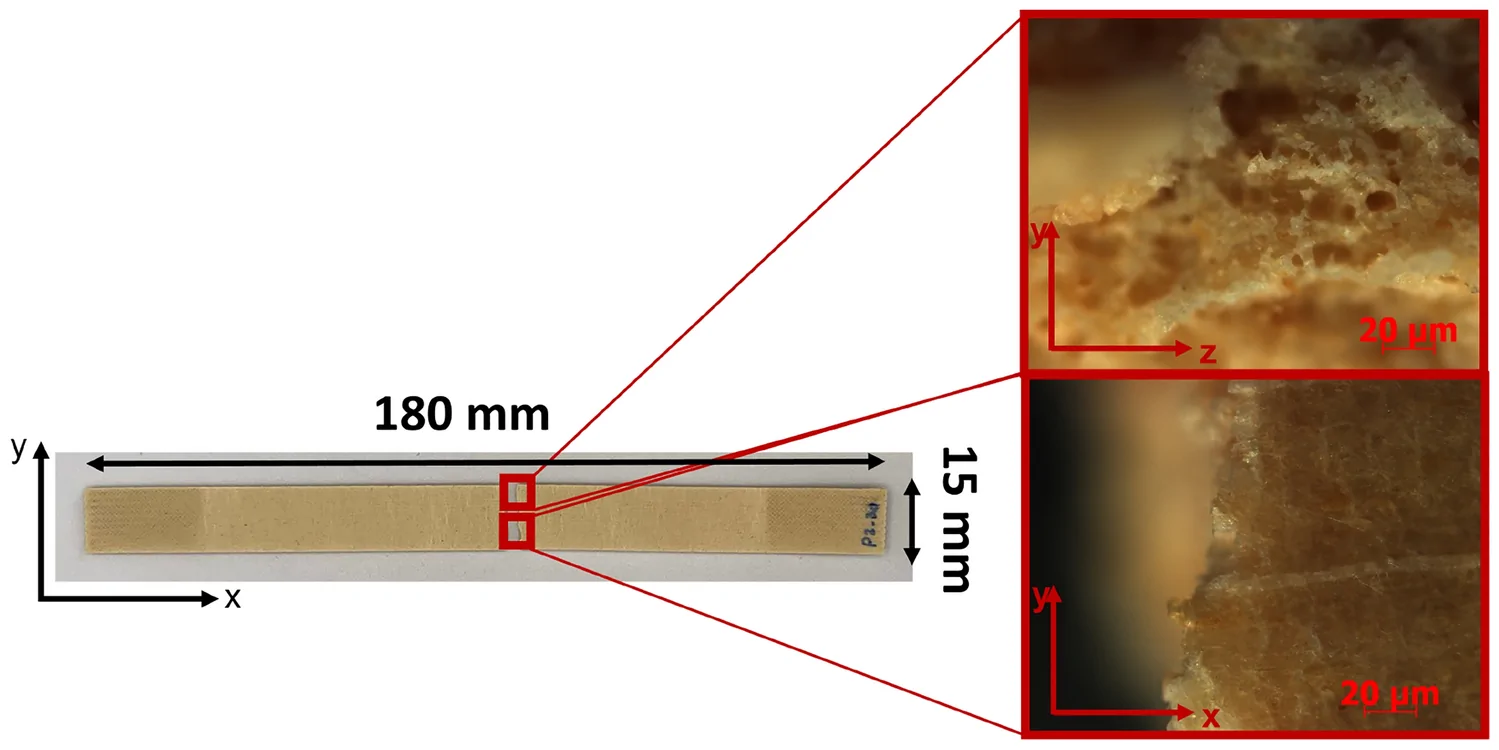
Optical microscopy observations of a fungal-colonised PLA_W30_WM specimen. The overview image identifies the investigated regions, while the detailed microscopic observations show the specimen cross-section (*y*–*z* plane) and surface region (*x*–*y* plane). Fungal structures were observed at both examination locations following the colonisation period. Scale bars correspond to 20 *μ*m.

Representative macroscopic and microscopic observations of fungal-colonised specimens are shown in Fig. 13. The highlighted regions were selected for microscopic examination after the two-week colonisation period. The microscopic observations reveal the presence of fungal structures on the specimen surface, confirming successful surface colonisation of the PLA/Wood specimens by *Fomes fomentarius*. Similar to the observations for PLA_W10 and PLA_W20, the present observations do not allow conclusions regarding the extent of fungal growth within the internal specimen structure.

Among the investigated commercial filament material combinations, PLA_W30 exhibited the highest measured values of mean *E* and mean UTS. The untreated PLA/Wood composite containing 30 wt.% wood-particle content reached a mean *E* of $2685.01 \pm 10.23~\mathrm {N/mm^2}$ and a mean UTS of $37.98 \pm 0.16~\mathrm {N/mm^2}$. Following fungal colonisation (PLA_W30_WM), the mean Young’s modulus decreased to $2409.32 \pm 9.70~\mathrm {N/mm^2}$, while the mean UTS decreased to $35.41 \pm 0.25~\mathrm {N/mm^2}$. These changes correspond to reductions of 10.3 % for mean *E* and 6.8 % for the mean UTS. Despite these reductions, PLA_W30_WM retained higher values of both mean *E* and mean UTS than all other investigated material combinations.

A one-way ANOVA followed by Tukey’s post-hoc test revealed statistically significant differences between PLA_W30 and PLA_W30_WM for both mean *E* (****p < 0.0001) and mean UTS (****p < 0.0001).

Despite the statistically significant reductions observed between the untreated and fungal-colonised specimen groups, PLA_W30_WM retained the highest measured values of both mean *E* and mean UTS among the investigated fungal-colonised material combinations. However, because the investigated materials originated from different commercial filaments, these results should be interpreted as material combination-specific observations rather than as evidence of an optimal wood-particle content.

#### PLA/Wood material combinations with 40 wt.% wood-particle content

**Fig. 14:**
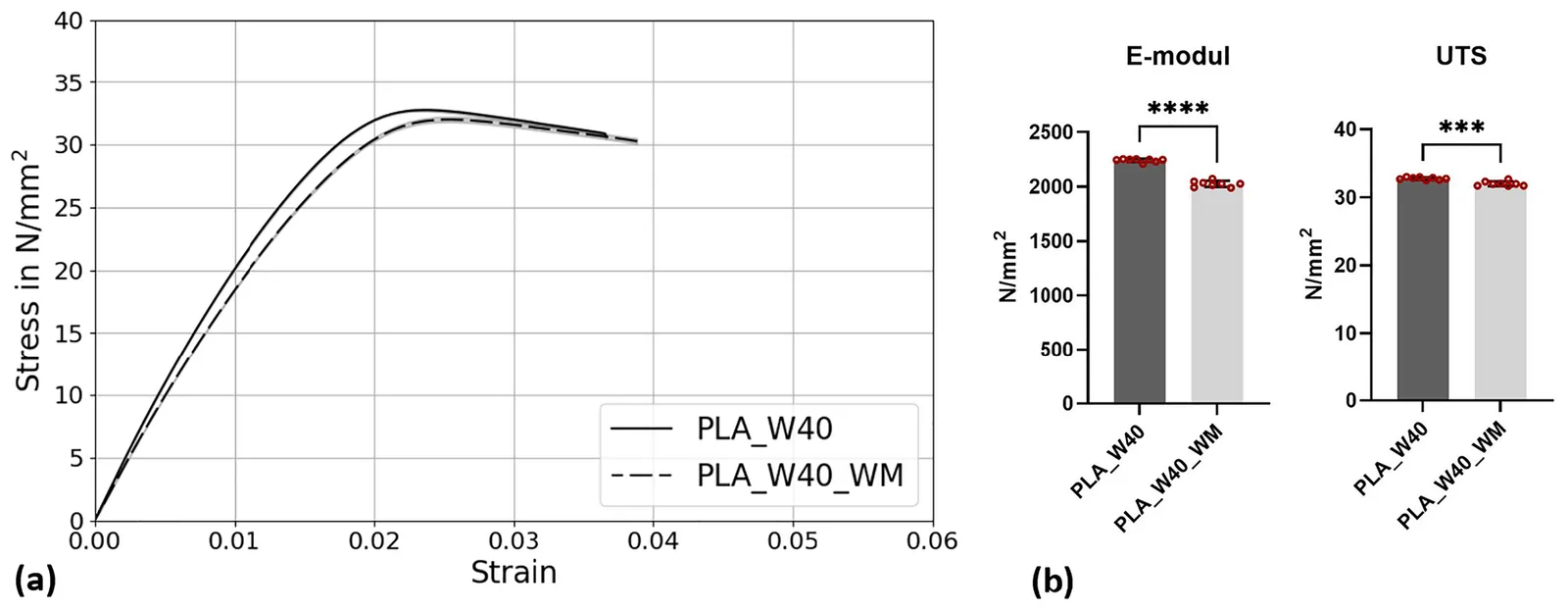
Mechanical characterisation of PLA_W40 and PLA_W40_WM specimens: (a) mean engineering stress–strain curves with corresponding 95 % confidence intervals and (b) comparison of mean *E* and mean UTS. Error bars represent standard deviations. Statistical significance was evaluated using one-way ANOVA followed by Tukey’s post-hoc test (*p<0.05, **p<0.01, ***p<0.001, ****p<0.0001).

Figure 14 summarises the mechanical response of both material combinations. Figure 14a presents the mean engineering stress–strain curves together with their 95 % confidence intervals, whereas Fig. 14b compares the resulting values of mean *E* and mean UTS. Both material combinations exhibited a similar overall tensile response, characterised by an initial approximately linear elastic region followed by a maximum stress level corresponding to the mean UTS. Compared with the untreated specimens, the fungal-colonised specimens exhibited a lower initial slope of the stress–strain curve and slightly lower maximum stress values, which is consistent with the reductions observed for both mean *E* and mean UTS.

**Fig. 15:**
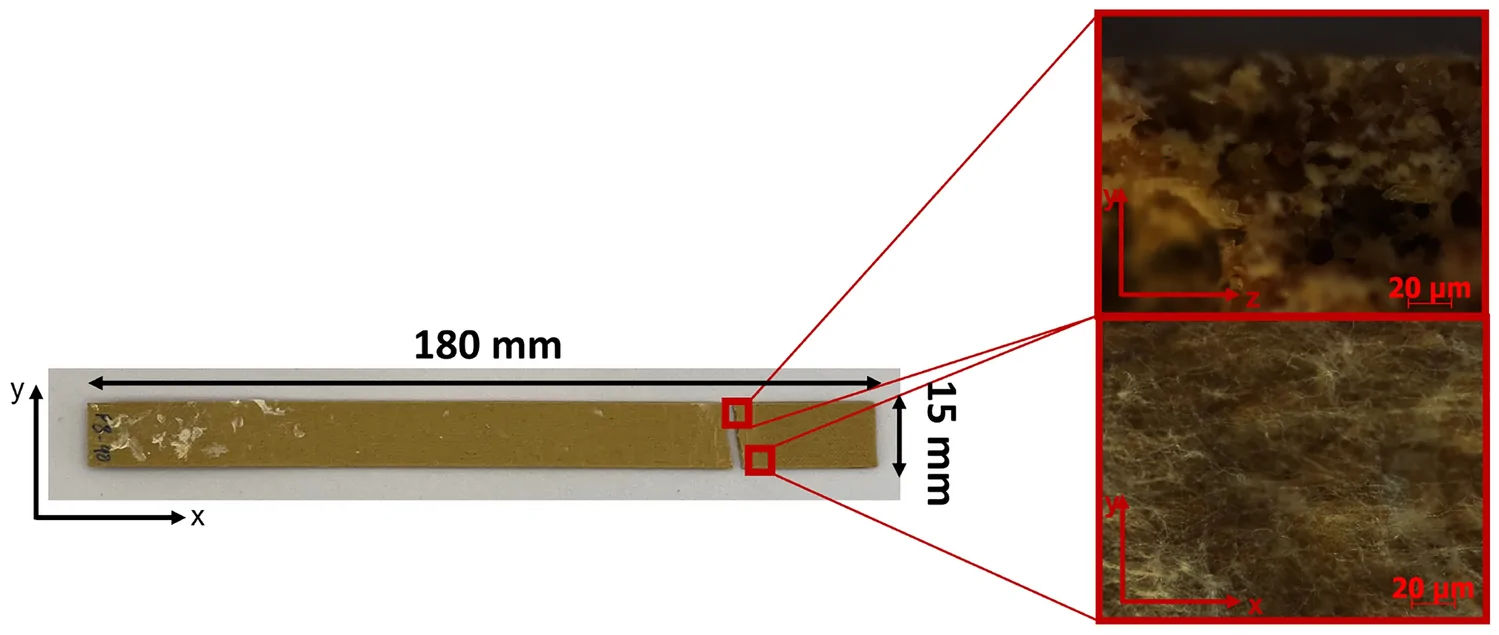
Representative microscopic examination of PLA_W40_WM specimens after two weeks of fungal colonisation. The marked positions in the macroscopic image indicate the analysed regions. The upper image depicts the cross-sectional area (*y*–*z* plane), whereas the lower image shows the specimen surface (*x*–*y* plane). Fungal structures are visible in both observation planes. Scale bars correspond to 20 *μ*m.

Representative macroscopic and microscopic observations of fungal-colonised specimens are shown in Fig. 15. The highlighted regions were selected for microscopic examination after the two-week colonisation period by *Fomes fomentarius*. The microscopic observations reveal the presence of fungal structures on the specimen surface, confirming successful surface colonisation of the PLA/Wood specimens by *Fomes fomentarius*. Similar to the observations for the lower wood-particle contents, the present observations do not allow conclusions regarding the extent of fungal growth within the internal specimen structure.

A further increase in wood-particle content to 40 wt.% resulted in lower mean *E* and mean UTS compared with the PLA_W30 material combination. The untreated PLA/Wood composite (PLA_W40) exhibited a mean *E* of $2242.16 \pm 5.04~\mathrm {N/mm^2}$ and a mean UTS of $32.78 \pm 0.07~\mathrm {N/mm^2}$. Following fungal colonisation (PLA_W40_WM), mean *E* decreased to $2025.39 \pm 9.14~\mathrm {N/mm^2}$, while the mean UTS decreased to $32.01 \pm 0.12~\mathrm {N/mm^2}$. These changes correspond to reductions of 9.7 % for mean *E* and 2.4 % for the mean UTS. Although both mean *E* and mean UTS decreased after fungal colonisation, the reduction in mean UTS was less pronounced than that observed for the lower wood-particle contents.

A one-way ANOVA followed by Tukey’s post-hoc test revealed statistically significant differences between PLA_W40 and PLA_W40_WM for both mean *E* (****p < 0.0001) and mean UTS (***p < 0.001).

The results obtained for PLA_W40 show differences in both stiffness and tensile strength between the untreated and fungal-colonised specimen groups. Compared with the values measured for PLA_W30, lower values of both mean *E* and mean UTS were observed for the untreated and fungal-colonised specimens. Nevertheless, the relative reduction in mean UTS remained comparatively small, suggesting that the tensile strength of this material combination was only moderately affected following the applied colonisation and associated processing conditions.

#### PLA/wood material combinations with 50 wt.% wood-particle content

**Fig. 16:**
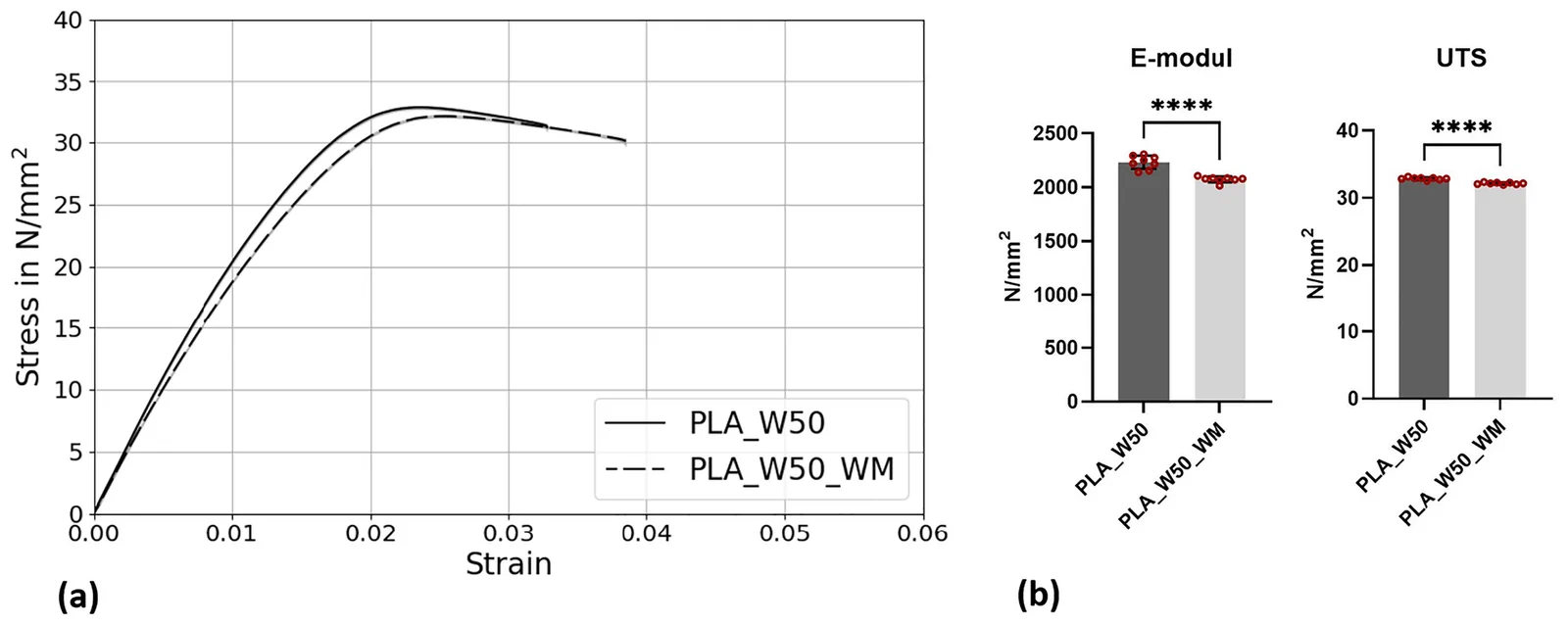
Mechanical characterisation of PLA_W50 and PLA_W50_WM specimens: (a) mean engineering stress–strain curves with corresponding 95 % confidence intervals and (b) comparison of mean *E* and mean UTS. Error bars represent standard deviations. Statistical significance was evaluated using one-way ANOVA followed by Tukey’s post-hoc test (*p<0.05, **p<0.01, ***p<0.001, ****p<0.0001).

Figure 16 summarises the mechanical response of both material combinations. Figure 16a presents the mean engineering stress–strain curves together with their 95 % confidence intervals, whereas Fig. 16b compares the resulting values of mean *E* and mean UTS. Both material combinations exhibited a similar overall tensile response, characterised by an initial approximately linear elastic region followed by a maximum stress level corresponding to the mean UTS. Compared with the untreated specimens, the fungal-colonised specimens exhibited a lower initial slope of the stress–strain curve and slightly lower maximum stress values, which is consistent with the reductions observed for both mean *E* and mean UTS.

**Fig. 17:**
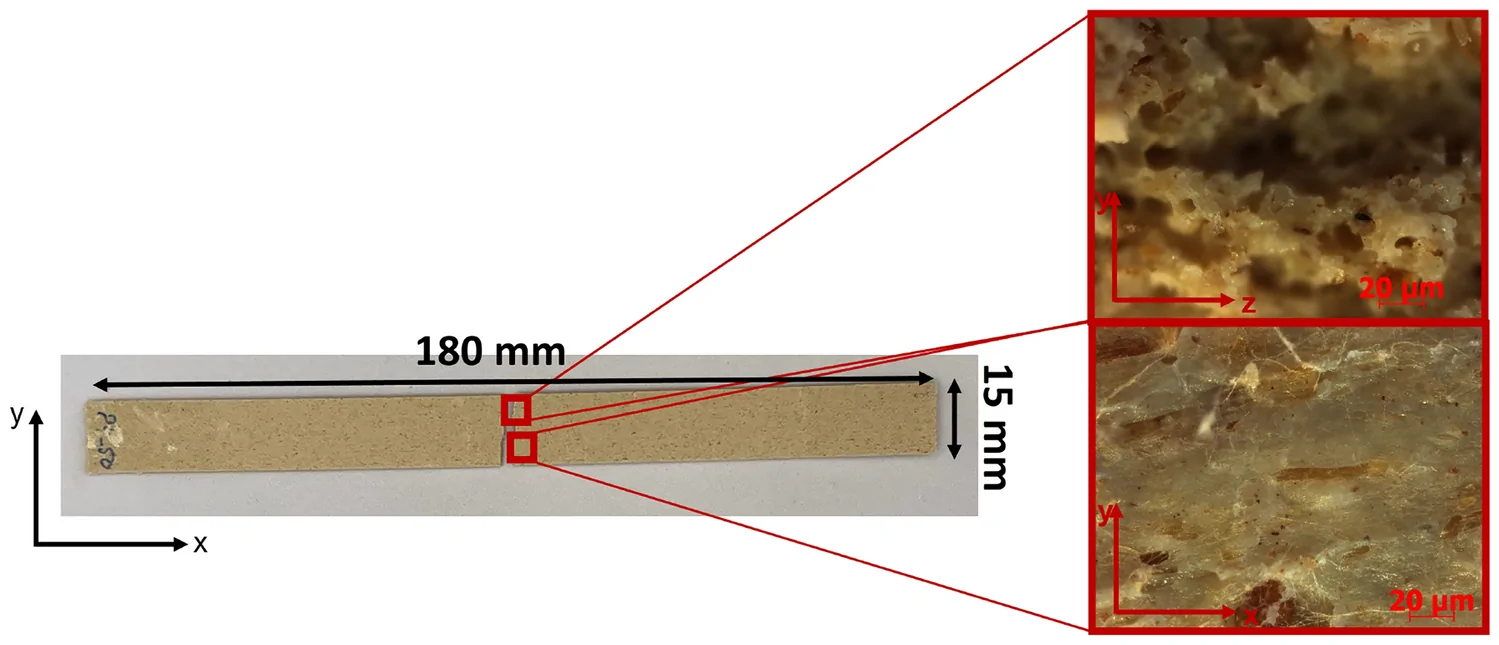
Macroscopic and microscopic characterisation of a fungal-colonised PLA_W50_WM specimen. The highlighted regions were selected for microscopy after colonisation with *Fomes fomentarius*. Representative images from the cross-section (*y*–*z* plane) and specimen surface (*x*–*y* plane) illustrate fungal structures present on the analysed specimen. Scale bars correspond to 20 *μ*m.

Representative macroscopic and microscopic observations of fungal-colonised specimens are shown in Fig. 17. The highlighted regions were selected for microscopic examination after the two-week colonisation period by *Fomes fomentarius*. The microscopic observations reveal the presence of fungal structures on the specimen surface, confirming successful surface colonisation of the PLA/Wood specimens by *Fomes fomentarius*. Similar to the observations for all other investigated material combinations, the present observations do not allow conclusions regarding the extent of fungal growth within the internal specimen structure.

At the highest investigated wood-particle content of 50 wt.%, the untreated PLA/Wood composite (PLA_W50) exhibited a mean *E* of $2231.05 \pm 20.33~\mathrm {N/mm^2}$ and a mean UTS of $32.86 \pm 0.07~\mathrm {N/mm^2}$. After fungal colonisation (PLA_W50_WM), the mean Young’s modulus decreased to $2072.72 \pm 8.80~\mathrm {N/mm^2}$, while the mean UTS decreased to $32.14 \pm 0.05~\mathrm {N/mm^2}$. These changes correspond to reductions of 7.1 % for mean *E* and 2.2 % for the mean UTS. Among all investigated fungal-colonised material combinations, PLA_W50_WM exhibited one of the smallest relative reductions in tensile strength.

A one-way ANOVA followed by Tukey’s post-hoc test revealed statistically significant differences between PLA_W50 and PLA_W50_WM for both mean *E* (****p < 0.0001) and mean UTS (****p < 0.0001).

For the highest investigated wood-particle content, statistically significant differences were still observed between untreated and fungal-colonised specimens for both mean *E* and mean UTS. However, the magnitude of the observed changes was comparatively small relative to the untreated reference specimens. Despite this, the measured tensile properties remained lower than those obtained for the PLA_W30 material combination. As discussed in Section 2.1, differences between the commercial filament systems may arise not only from nominal wood-particle content but also from manufacturer-specific material compositions and processing routes.

### Statistical evaluation of mean young’s modulus and mean UTS

**Fig. 18:**
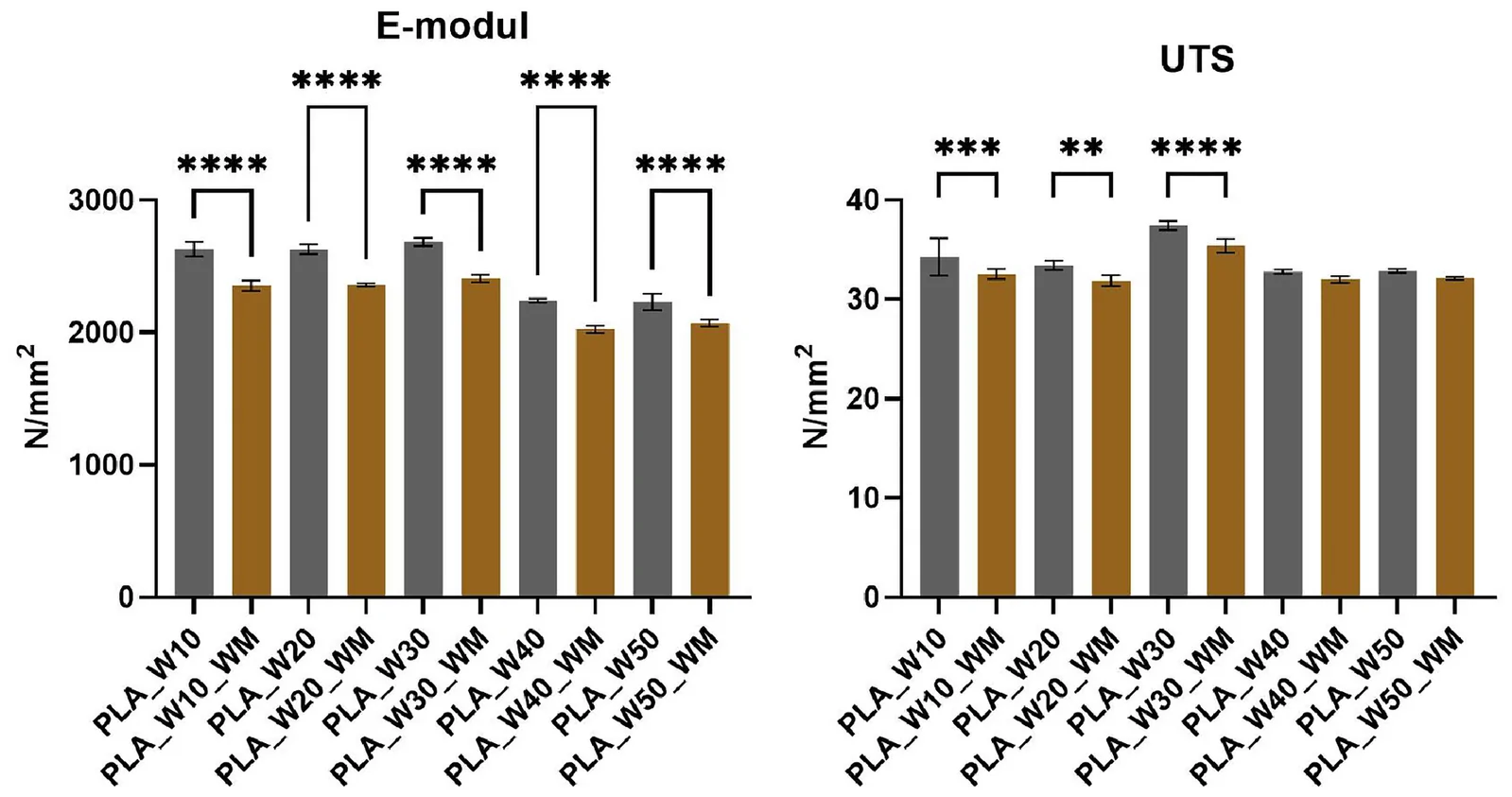
Statistical comparison of mean *E* and mean UTS for all investigated PLA/wood material combinations before and after fungal colonisation. Grey bars represent untreated specimens, whereas brown bars represent fungal-colonised specimens (WM). Error bars indicate standard deviations. Statistical significance was evaluated using one-way ANOVA followed by Tukey’s post-hoc test (*p<0.05, **p<0.01, ***p<0.001, ****p<0.0001).

Figure 18 summarises the statistical comparison of mean *E* and mean UTS for all investigated PLA/wood material combinations before and after fungal colonisation.

The results demonstrate that statistically significant differences in mean Young’s modulus were observed between untreated and fungal-colonised specimens for all investigated wood-particle contents. Similarly, statistically significant reductions in mean UTS were observed for all material combinations, although the magnitude of the effect varied between material combinations.

Among the investigated commercial filament material combinations, PLA_W30 and PLA_W30_WM exhibited the highest measured values of both mean *E* and mean UTS, confirming the trends previously observed in the stress–strain analyses. Furthermore, the statistical evaluation confirms that significant differences exist between the investigated material combinations. However, because the commercial filaments originated from different manufacturers, these differences cannot be attributed exclusively to the nominal wood-particle content.

### LCA results based on life cycle inventory data

Table 5 summarises the life cycle inventory data used as inputs to the supplementary screening-level printing-stage LCA for the five investigated PLA/wood material combinations (*w = 0.5* to 0.1). For each material combination, the foreground inventory includes the calculated electricity consumption $E_{\textrm{el}}$ and the measured total specimen mass $m_{\textrm{tot}}$. The corresponding PLA and wood masses are derived from $m_{\textrm{tot}}$ according to the nominal wood-particle mass fraction.

Across the five material combinations, electricity consumption remains within a narrow range of 0.023–0.024 kWh per specimen. The total specimen mass varies from 6.030 to 6.684 g. With decreasing wood-particle content, the calculated PLA mass increases from 3.1200 to 6.0156 g, while the calculated wood mass decreases from 3.1200 to 0.6684 g.

**Table 5:** Inventory data used as inputs for the supplementary printing-stage LCA. The wood-particle content *w* is the nominal wood-particle mass fraction (0–1 by mass). PLA and wood masses are derived from $m_{\textrm{PLA}}=(1-w)m_{\textrm{tot}}$ and $m_{\textrm{wood}}=wm_{\textrm{tot}}$.

Sample ID	*w* (-)	Total mass (g)	PLA mass (g)	Wood mass (g)	Electricity (kWh)	Notes
1	0.50	6.240	3.1200	3.1200	0.024	50 % wood
2	0.40	6.030	3.6180	2.4120	0.024	40 % wood
3	0.30	6.260	4.3820	1.8780	0.023	30 % wood
4	0.20	6.600	5.2800	1.3200	0.023	20 % wood
5	0.10	6.684	6.0156	0.6684	0.023	10 % wood

**Fig. 19:**
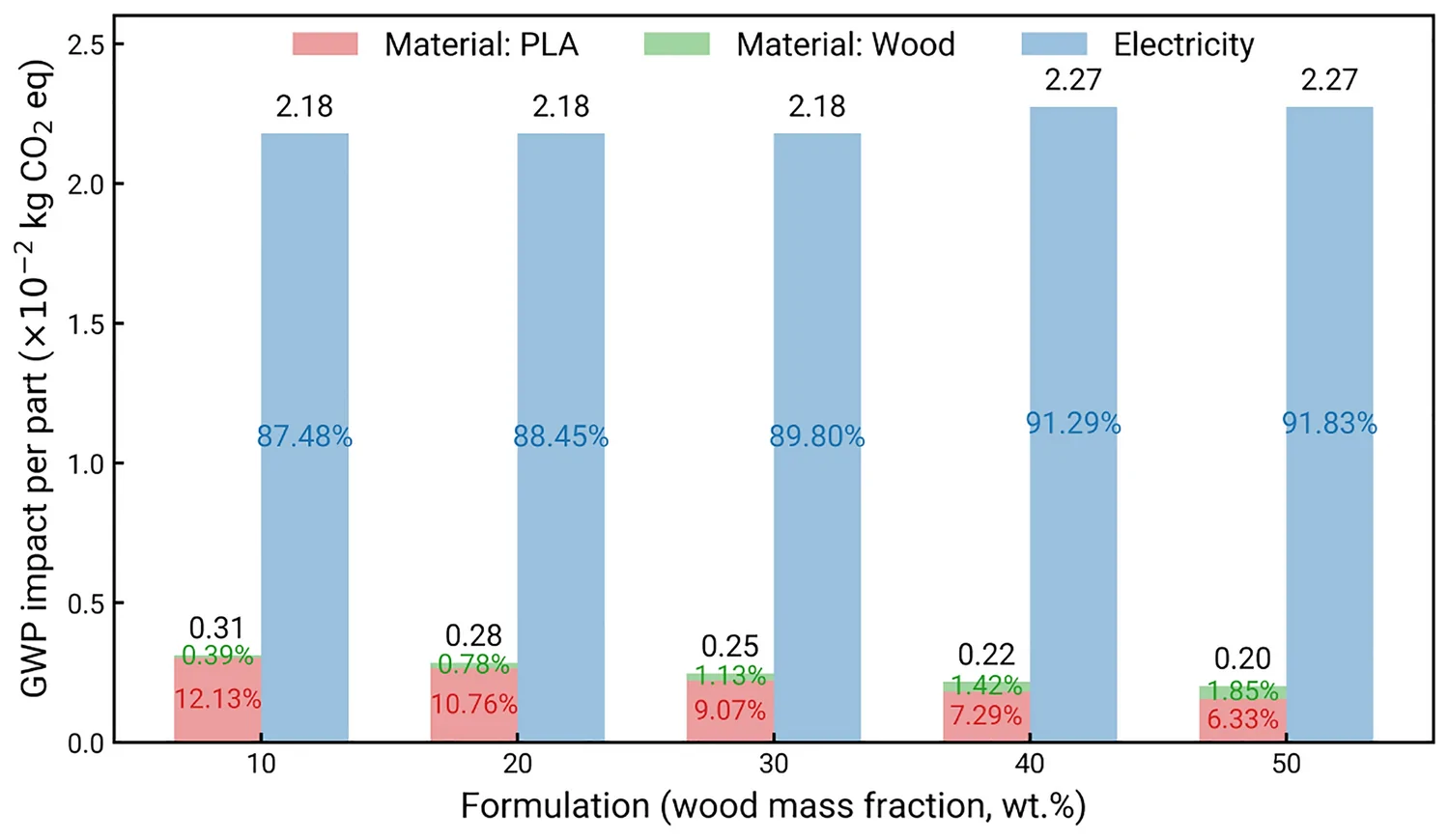
Contribution breakdown for GWP for the investigated PLA/wood material combinations. The total impact is decomposed into electricity-related and material-related contributions, with the material term further split into PLA- and wood-attributed components.

Figure 19 presents the contribution breakdown for GWP. The total GWP remains within a narrow range of 0.02426–0.02491 kg CO$_2$ eq per specimen across the five PLA/wood material combinations. Sample 3 (*w=0.30*) shows the lowest total GWP, whereas Samples 2 and 5 show the highest total GWP values. In all material combinations, the electricity-related contribution forms the dominant baseline, ranging from 0.02179 to 0.02274 kg CO$_2$ eq per specimen. The material-related contribution is smaller, ranging from 0.00202 to 0.00312 kg CO$_2$ eq per specimen, and varies with the PLA/wood mass split.

As the nominal wood-particle content decreases from 50 % to 10 %, the PLA-related GWP contribution increases from 0.00157 to 0.00302 kg CO$_2$ eq, while the wood-related contribution decreases from 0.00046 to 0.00010 kg CO$_2$ eq. Therefore, the variation in material-related GWP is mainly associated with the increasing PLA mass fraction. However, because the electricity-related contribution is substantially larger than the material-related contribution, the total GWP values remain comparatively close across the five material combinations.

The corresponding contribution breakdowns for AP and EP, together with the detailed numerical LCA results, are provided in Appendix A. These supplementary results show that AP and EP vary more clearly with decreasing wood-particle content than GWP.

## Discussion

The presented results provide a comprehensive overview of the influence of fungal colonisation on the tensile behaviour of additively manufactured PLA/wood material combinations. Based on these findings, the mechanical research question defined in Section 1 can now be addressed.

### Influence of fungal colonisation on mechanical response (RQ1)

Across all investigated material combinations, the fungal-colonised specimens exhibited statistically significantly lower mean *E* and mean UTS values than the corresponding untreated specimens. The reduction in mean Young’s modulus ranged from approximately 7.1 % to 10.5 %, while the reduction in mean UTS ranged from approximately 2.2 % to 6.8 %. Although the magnitude of the reductions varied between material combinations, the overall trend was consistent across all investigated PLA/wood material combinations.

The statistical evaluation presented in Fig. 18 supports these observations. For Young’s modulus, highly significant differences (*p<0.0001*) were identified between untreated and fungal-colonised specimens for all investigated material combinations. Similarly, statistically significant reductions in mean UTS were observed for all material combinations, confirming that the measured differences are unlikely to result from random experimental variation alone.

The stress–strain curves further demonstrated that fungal-colonised specimens generally exhibited lower initial slopes and lower maximum stress values compared with their untreated counterparts. Since mean Young’s modulus was determined from the initial linear region of the stress–strain curves, the observed reductions indicate a decrease in tensile stiffness in the fungal-colonised specimen groups. Likewise, the reduction in mean UTS demonstrates that fungal colonisation also affected the maximum load-bearing capacity of the investigated material combinations.

A pronounced stress peak followed by a distinct post-peak softening region was particularly observed for the untreated and fungal-colonised 40 wt.% wood-particle material combinations (PLA_W40 and PLA_W40_WM). In both cases, the stress increased rapidly to a maximum value before decreasing towards a more stable plateau region at higher strain levels. Since no in-situ damage monitoring was performed during tensile testing, the mechanisms responsible for this behaviour cannot be identified directly. However, the pronounced peak indicates that local damage initiation and load redistribution processes may occur shortly after the maximum stress level is reached. Additional investigations combining mechanical testing with advanced microstructural characterisation would be required to establish the origin of this characteristic stress–strain response.

Microscopic observations confirmed successful surface colonisation of all investigated material combinations by *Fomes fomentarius*. Hyphal structures were observed on specimen surfaces and within surface-adjacent regions after incubation. However, the present study does not allow direct conclusions regarding the underlying mechanisms responsible for the observed reductions in mechanical performance. While fungal growth was clearly visible on the specimen surfaces, additional investigations using techniques such as scanning electron microscopy (SEM), micro-computed tomography (*μ*CT), or interfacial characterisation would be required to establish direct relationships between fungal colonisation and changes in the internal composite structure.

An additional limitation of the present study concerns the potential influence of the fungal colonisation protocol itself on the mechanical response of the investigated PLA/wood material combinations. Although successful fungal growth was confirmed by microscopy, fungal hyphae were primarily observed on the specimen surfaces and within surface-adjacent regions. Furthermore, no sham-treated control specimens were included to isolate the individual effects of incubation, moisture exposure, drying, and specimen handling. Following fungal colonisation, all specimens were oven-dried at 55 $^\circ$C and subsequently vacuum sealed prior to mechanical testing. Nevertheless, the moisture content of the specimens was not quantitatively determined before or after colonisation, and the relative humidity during the incubation period was not monitored. Consequently, the observed reductions in mean Young’s modulus (*E*) and mean ultimate tensile strength (UTS) should be interpreted as the mechanical response of specimens subjected to the fungal colonisation protocol rather than being attributed exclusively to fungal activity. Future investigations should therefore include sham-treated controls together with quantitative moisture measurements to distinguish moisture-related effects from the direct influence of fungal colonisation.

An additional limitation of the present study concerns the potential influence of moisture introduced during the fungal colonisation process. PLA and lignocellulosic fillers are known to exhibit hygroscopic behaviour, and moisture uptake may affect the mechanical response of PLA/wood composites. Following fungal colonisation, all specimens were oven-dried at $55^\circ$C to terminate fungal activity and subsequently vacuum sealed prior to mechanical testing. However, the moisture content of the specimens was not quantitatively determined before or after colonisation, and the relative humidity during the incubation period was not monitored. Consequently, the observed reductions in mean Young’s modulus (*E*) and mean ultimate tensile strength (UTS) cannot be attributed exclusively to fungal colonisation, as moisture-related effects may also have contributed to the measured mechanical response. Future investigations should therefore include quantitative moisture measurements and moisture-controlled reference specimens to distinguish between moisture-related and biological effects.

This interpretation is consistent with our previous work^14^, in which specimens subjected to a fungal cultivation protocol likewise exhibited measurable changes in mechanical behaviour. As discussed in that study, environmental conditions associated with the cultivation process, including incubation and moisture exposure, may also contribute to changes in mechanical properties. Related studies on mycelium-based material combinations have similarly demonstrated that fungal cultivation can alter the structural characteristics and mechanical response of bio-based material combinations depending on the fungal species, substrate composition, and cultivation conditions^43^. Although direct quantitative comparisons between studies remain challenging due to differences in material combinations, specimen geometries, incubation conditions, and testing methodologies, the available literature collectively indicates that fungal cultivation protocols can influence the performance of engineered composite materials.

It should further be noted that the investigated PLA/wood filaments originated from different commercial manufacturers and may therefore differ not only in nominal wood-particle content but also in PLA grade, additives, wood species, particle morphology, and compounding procedures. Consequently, differences observed between individual material combinations cannot be attributed exclusively to the nominal wood-particle content. Nevertheless, the influence of fungal colonisation remained consistent across all investigated material combinations.

Accordingly, the mechanical research question can be answered by concluding that post-printing fungal colonisation by *Fomes fomentarius* consistently reduced both mean *E* and mean UTS of the investigated PLA/wood material combinations. The statistically significant differences identified by the ANOVA analyses, together with the observed changes in the stress–strain response and the confirmed presence of fungal growth on the specimen surfaces, provide clear evidence that fungal colonisation influenced the tensile behaviour of the investigated materials.

### Environmental implications of the investigated PLA/wood material combinations (RQ2)

The supplementary screening-level LCA provides an environmental interpretation of the investigated PLA/wood material combinations at the MEX AM printing stage. Within the defined gate-to-gate boundary, the environmental profile is mainly determined by two foreground contributions: electricity consumption during printing and material-combination-dependent material consumption. This separation between electricity- and material-related contributions is informed by the previously published XAI–LCA workflow, in which printing-stage impacts are interpreted through energy- and material-driven pathways^53^. Similar distinctions between process energy demand and material consumption are also discussed in LCA studies of MEX AM processes^59,60^.

For GWP, the most important outcome is that the total impact is dominated by the electricity-related contribution. Since all investigated specimens are produced under comparable printing conditions and show only small differences in calculated electricity consumption, the electricity term forms a nearly constant baseline across the five PLA/wood material combinations. The PLA/wood mass split changes the material-related contribution, but this effect is superimposed on the larger electricity-related baseline. Therefore, changing the wood-particle content alone has only a limited influence on total GWP within the present printing-stage boundary. This result is in line with previous MEX AM LCA studies in which process energy demand is identified as an important contributor to climate-related impacts^53,60^.

AP and EP provide a different environmental perspective. In contrast to GWP, these indicators are more sensitive to the material composition of the printed specimen. When the PLA fraction increases, the material-related AP and EP contributions become more pronounced. This trend reflects the different cradle-to-gate characterization factors assigned to PLA resin and wood flour in the present screening model^56–58^. Similar findings in LCA studies of PLA-based and wood-fibre-reinforced biocomposites show that the environmental interpretation of bio-based composites depends strongly on the selected impact category and should not be reduced to climate impact alone^61^. Therefore, AP and EP are retained as supplementary indicators because they reveal material-combination-dependent effects that are less visible in the GWP results.

These findings address RQ2 by showing that the environmental implications of the investigated PLA/wood material combinations are category-specific. GWP mainly reflects the electricity demand of the printing process, whereas AP and especially EP are more sensitive to the PLA/wood material split. A higher wood-particle content can reduce the PLA-related material contribution, but the environmental benefit depends on the selected midpoint indicator and on the printing-stage electricity demand. Therefore, the LCA results should be understood as an environmental profile of the investigated material combinations rather than as a single universal ranking. The relationship between these environmental findings and the mechanical performance of the printed specimens is discussed in the following section.

### Relationship between mechanical performance and environmental impacts

The supplementary screening-level Life Cycle Assessment (LCA) provides additional insight into the environmental implications of the investigated PLA/wood material combinations and complements the mechanical characterisation presented in Sections 3.1 and 3.2.

The contribution analysis demonstrated that the environmental impacts of the investigated material combinations were governed by different mechanisms depending on the selected impact category. For GWP, electricity consumption during the MEX AM process represented the dominant contribution, accounting for approximately 88–92 % of the total impact. Consequently, variations in wood-particle content resulted in only relatively small differences in total GWP because all specimens were manufactured using nearly identical printing conditions and exhibited similar electricity consumption values.

In contrast, AP and EP were more strongly influenced by material composition. The contribution analysis showed that increasing wood-particle content reduced the relative amount of PLA required per specimen and therefore decreased the material-related environmental burdens. This effect was particularly pronounced for EP, where the material contribution represented approximately 95–98 % of the total impact. For AP, both electricity consumption and material composition contributed substantially to the overall results.

When the mechanical and environmental results are considered together, a trade-off becomes apparent. Fungal colonisation consistently reduced both mean *E* and mean UTS, as confirmed by the ANOVA results presented in Figure 18. At the same time, increasing wood-particle content generally reduced material-related environmental impacts by partially replacing PLA with a lignocellulosic filler associated with lower cradle-to-gate impacts.

However, the mechanical results indicate that increasing wood-particle content does not automatically lead to improved tensile performance. Although the investigated commercial filament combinations containing approximately 30 wt.% wood particles exhibited the highest measured values of mean *E* and UTS, direct comparison between all investigated materials remains limited because the filaments originated from different manufacturers and may differ in PLA grade, additives, wood species, particle morphology, and compounding procedures. Consequently, the observed trends should be interpreted as material-combination-specific behaviour rather than as evidence for an optimal wood-particle content.

Overall, within the assumptions of the present screening-level assessment, increasing wood-particle content can reduce material-related environmental burden indicators, while fungal colonisation introduces a measurable reduction in tensile performance. The results therefore highlight the importance of simultaneously considering mechanical requirements and environmental impacts when selecting PLA/wood material combinations for additively manufactured applications.

## Conclusion

This study investigated the mechanical response associated with nominal wood-particle content and post-printing fungal colonisation by *Fomes fomentarius*  in the tensile behaviour of additively manufactured PLA/wood material combinations manufactured by MEX AM. In addition, a screening-level Life Cycle Assessment (LCA) was performed to evaluate environmental impacts associated with the fabrication stage.

The main findings can be summarised as follows:

- The investigated PLA/wood material combinations exhibited mean Young’s modulus (*E*) values between  2025.39 and 2685.01 $\mathrm {N/mm^2}$ and mean Ultimate Tensile Strength (UTS) values between 31.88 and 37.98 $\mathrm {N/mm^2}$.
- Because the investigated commercial filaments originated from different manufacturers, differences in mechanical performance cannot be attributed exclusively to nominal wood-particle content and may additionally be influenced by formulation-specific factors such as PLA grade, additives, wood species, particle morphology, and compounding procedures.
-  Across all investigated material combinations, fungal-colonised specimens exhibited lower mechanical performance than their corresponding untreated counterparts. Mean *E* decreased by approximately 7.1–10.5 %, while mean UTS decreased by approximately 2.2–6.8 %. ANOVA analyses confirmed statistically significant differences between untreated and fungal-colonised specimens.
- Microscopy images confirmed successful fungal growth on specimen surfaces. However, the present study does not allow direct conclusions regarding the mechanisms responsible for the observed reductions in mean *E* and mean UTS, highlighting the need for further microstructural investigations.
- Within the present screening-level assessment, the relative importance of material composition and manufacturing energy depends on the considered impact category. GWP is dominated by electricity consumption during MEX AM fabrication, EP is primarily governed by PLA content, and AP is influenced by both material consumption and electricity demand.

Overall, the combined mechanical and environmental evaluation demonstrates that fungal colonisation can significantly affect the tensile performance of additively manufactured PLA/wood material combinations, while environmental impacts depend on different underlying mechanisms. Consequently, mechanical performance and environmental performance should be considered simultaneously when assessing bio-based additively manufactured composites.

The presented framework combining tensile characterisation, fungal colonisation, microscopy, statistical analysis, and screening-level LCA provides a basis for future investigations of sustainable fungal-mycelium-colonised additively manufactured composite materials.

## Supplementary Information

Supplementary Information 1.

Supplementary Information 2.

Supplementary Information 3.

Supplementary Information 4.

Supplementary Information 5.

Supplementary Information 6.

## Data Availability

Data for the additive manufacturing specimens presented in this work are available on GitHub: https://github.com/SVFS-TUBerlin/Mechanical-characterization-of-biopolymer-reinforced-composites Raw experimental data can be provided upon request from the corresponding author. Code availability: The Python code underlying the previously published XAI-assisted LCA workflow referenced in this study is publicly available at: https://github.com/SVFS-TUBerlin/Publications_Supplementary_Materials/tree/main/2026_Zhu_VPP_XAI_LCA_MEX

## Materials availability

All materials used in the study are commercially available or described within the manuscript. No new materials were developed for this study.

## Code availability

The Python code underlying the previously published XAI-assisted LCA workflow referenced in this study is publicly available at https://github.com/SVFS-TUBerlin/Publications_Supplementary_Materials/tree/main/2026_Zhu_VPP_XAI_LCA_MEX.
