# Supplementary material for: Tensile characterization and life cycle assessment of fungal-colonized 3D-printed PLA/wood biocomposites
Source: Sci Rep. 2026 Aug 22;16:26365. doi: 10.1038/s41598-026-65923-5 (PMC13499799; doi:10.1038/s41598-026-65923-5)
Supplement: Supplementary file 1 [file 41598_2026_65923_MOESM1_ESM.pdf]

A Supplementary LCA Results

Detailed numerical results of the supplementary screening-level printing-stage LCA are reported in Table 6.

Table 6: Screening-level printing-stage LCA results per printed specimen for five PLA/Wood material combinations (Samples 1–5). Results are decomposed into material-related contributions (PLA and wood) and electricity-related contributions (openLCA/TRACI), and summed linearly.

**Category**		**Unit**	**1**	**2**	**3**	**4**	**5**
**PLA contribution (material)**							
GWP	Global Warming Potential	kg CO_2 eq	0.001566	0.001816	0.002200	0.002651	0.003020
AP	Acidification Potential	kg SO_2 eq	0.000066	0.000076	0.000092	0.000111	0.000126
EP	Eutrophication Potential	kg N eq	0.000041	0.000048	0.000058	0.000070	0.000080
**Wood contribution (material)**							
GWP	Global Warming Potential	kg CO_2 eq	0.000457	0.000353	0.000275	0.000193	0.000098
AP	Acidification Potential	kg SO_2 eq	0.000005	0.000004	0.000003	0.000002	0.000001
EP	Eutrophication Potential	kg N eq	0.000000	0.000000	0.000000	0.000000	0.000000
**Total material impact (PLA + wood)**							
GWP	Global Warming Potential	kg CO_2 eq	0.002023	0.002169	0.002475	0.002844	0.003118
AP	Acidification Potential	kg SO_2 eq	0.000070	0.000080	0.000095	0.000113	0.000127
EP	Eutrophication Potential	kg N eq	0.000042	0.000048	0.000058	0.000070	0.000080
**Electricity contribution (per part)**							
GWP	Global Warming Potential	kg CO_2 eq	0.022735	0.022735	0.021787	0.021787	0.021787
AP	Acidification Potential	kg SO_2 eq	0.000056	0.000056	0.000053	0.000053	0.000053
EP	Eutrophication Potential	kg N eq	0.000002	0.000002	0.000002	0.000002	0.000002
**Total impact (material + electricity)**							
GWP	Global Warming Potential	kg CO_2 eq	0.024757	0.024904	0.024262	0.024631	0.024905
AP	Acidification Potential	kg SO_2 eq	0.000126	0.000135	0.000148	0.000166	0.000181
EP	Eutrophication Potential	kg N eq	0.000044	0.000050	0.000060	0.000072	0.000082

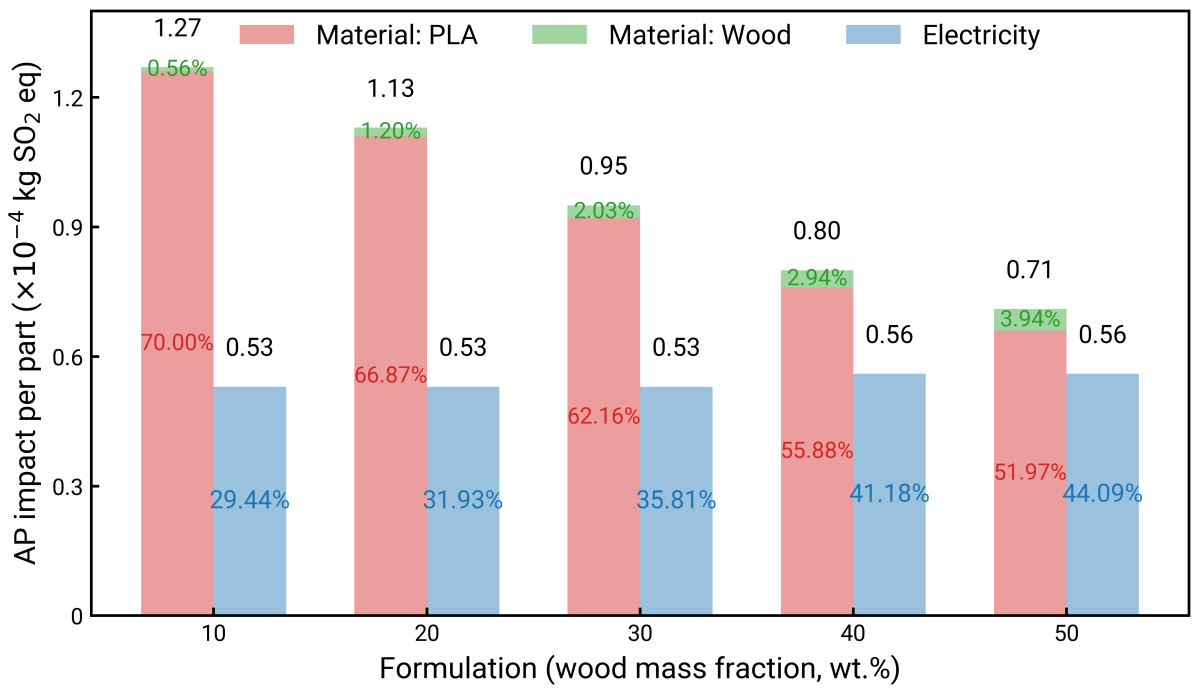

(a) AP contribution structure (material split into PLA/-wood vs. electricity).

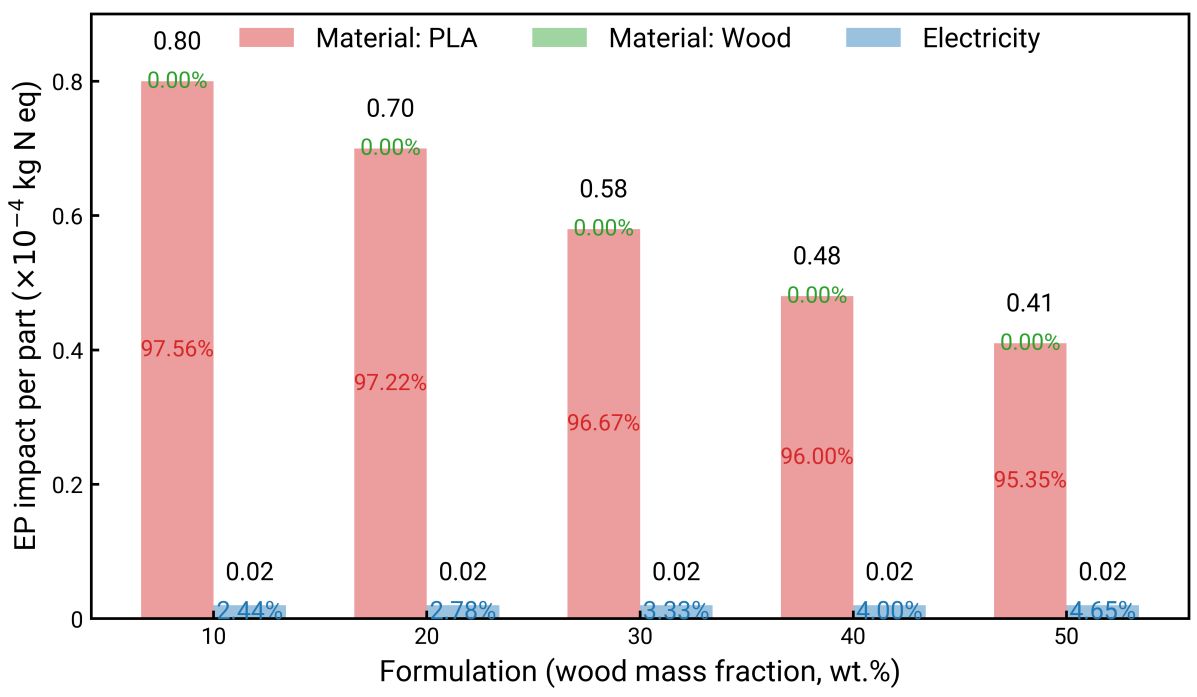

(b) EP contribution structure (material split into PLA/-wood vs. electricity).

Figure 20: Supplementary contribution plots for Acidification Potential (AP) and Eutrophication Potential (EP) for the investigated PLA/Wood formulations. In both cases, the total impact is decomposed into electricity-related and material-related contributions, with the material term further split into PLA- and wood-attributed components.
