# Supplementary material for: Tensile characterization and life cycle assessment of fungal-colonized 3D-printed PLA/wood biocomposites
Source: Sci Rep. 2026 Aug 22;16:26365. doi: 10.1038/s41598-026-65923-5 (PMC13499799; doi:10.1038/s41598-026-65923-5)
Supplement: Supplementary file 3 [file 41598_2026_65923_MOESM3_ESM.pdf]

Technical Data Sheet

(Printed, non-injection molded)

Product name: **3DJAKE ecoPLA Wood**
Version: 1.1
Date: 01.01.2024

Dimensions

Size	Ø tolerance	Roundness
**1,75 mm**	± 0,05 mm	≥ 95%

Physical properties

Description	Typical value	Test method
Specific gravity	1,1128 g/cc	ISO 1183
MFR 210 °C/2,16kg	5,36 g/10 min	ASTM-D-1238
Tensile strength at Yield	24,617 MPa	ISO 527
Strain at yield	7,078%	ISO 527
Strain at break	23,824%	ISO 527
E-Modulus	369,993 MPa	ISO 527
Impact strength	3,244 kJ/ m^2	ISO 179

Thermal properties

Description	Typical value	Test method
Printing temperature	210 – 230 °C	-

Packaging:
All spools are sealed and packed with silica gel to avoid humidity.

Additional info:
Due to its low tendency to warp 3DJAKE ecoPLA Wood can also be printed without a heated bed.
If you have a heated bed the recommended temperature is ± 35-60˚C.
PLA can be used on all common desktop FDM or FFF technology 3D printers.

Storage: Cool and dry (15-25˚C) and away from UV light. This enhances the shelf life significantly.

Web: www.3djake.com
Mail: office@3djake.com

Niceshops GmbH
Saaz 99, Paldau
Austria
