# Supplementary material for: Tensile characterization and life cycle assessment of fungal-colonized 3D-printed PLA/wood biocomposites
Source: Sci Rep. 2026 Aug 22;16:26365. doi: 10.1038/s41598-026-65923-5 (PMC13499799; doi:10.1038/s41598-026-65923-5)
Supplement: Supplementary file 4 [file 41598_2026_65923_MOESM4_ESM.pdf]

Wuhu R3D Technology Co., Ltd.
Add: Fanchang Economic Development Zone, Wuhu, Anhui,China
Tel: 0086-057488037579
Web: www.r3dprinter.com
Email: sales@china3dprinter.cn

Technical Data Sheet

R3D WOOD FILAMENT

Date: 10.11.2021

Dimensions	
Size	1,75 mm
Tolerance	± 0,02 mm
Thermal Features	
Printing Temperature	190-210℃
Melting Temperature	150-170℃
Platform Temperature	45-60℃
**Physical Features**	
Specific Gravity	1.2g/cm³
MFR 190℃/2,16kg	6g/10min
Tensile Strength at Yield	36.7 MPa
Strain At Break	6%
Flexural Strength	68.6 MPa
Flexural Modulus	3050 MPa
Impact Strength	0.3-0.5%
Moisture Absorption	<0.5
Packing	
Packing Method	Vacuum Sealing + Desiccant
Color Box Size	21.5*21.5*7CM
Net Weight	1000g
Gross Weight	1350g
Storage	
Cool and dry (15-25˚C) and away from direct sun light. This will enhance the shelf life.	
