# Supplementary material for: Tensile characterization and life cycle assessment of fungal-colonized 3D-printed PLA/wood biocomposites
Source: Sci Rep. 2026 Aug 22;16:26365. doi: 10.1038/s41598-026-65923-5 (PMC13499799; doi:10.1038/s41598-026-65923-5)
Supplement: Supplementary file 5 [file 41598_2026_65923_MOESM5_ESM.pdf]

Technical Data Sheet

Product name: EasyWood™

Date of issue: 29 October 2015
Version: v2

EasyWood™ looks, feels and smells like real wood. EasyWood™ is a unique mixture of 40% grinded wood particles in combination with the modified and proven easy-to-print binding polymers used for our EasyFil™ PLA filaments. This extraordinary blend has resulted in a high-end and unsurpassed "easy to print" wooden filament.
You can obtain a beautiful wood-nerve structure in your EasyWood™ printed objects by grinding it a little bit with a piece of fine grit sandpaper. By grinding your printed object a bit you will highlight the relief, or rough edges/surfaces, of your printed object with different shades of the respective wood colour.

Properties	Typical value	Test Method	Test condition
**Physical**			
Specific gravity	1.20 g/cc	ASTM D1505	-
Melt flow rate	4.5 g/10min	-	-
Water absorption	-	-	-
Moisture absorption	-	-	-
**Mechanical**			
Impact strength	7.1 KJ/m²	-	-
Tensile strength	71 Mpa (MD)	ASTM D882	-
Tensile modulus	1930 Mpa (MD)	ASTM D882	-
Elongation at break	171% (MD)	ASTM D882	-
Flexural strength	± 59.6 Mpa	-	-
Flexural modulus	± 2583.9 Mpa	-	-
Hardness	-	-	-
**Thermal**			
Print temperature	± 200 - 240° C	-	-
Melting termperature	± 145 ± 10° C	-	-
Viscat softening temp.	± 46° C	ISO 306	-
**Optical**			
Haze	-	-	-
Transmittance	-	-	-
Gloss	-	-	-

Product details, certifications and compliance	
HS Code	39169090
REACH compliant	Yes
RoHS certified	Yes
FDA compliant	No

Diameter	Tolerance	Roundness
1.75mm	± 0.05mm	≥ 95%
2.85mm	± 0.10mm	≥ 95%

Formfutura VOF
Groenestraat 215
6531 HH Nijmegen
The Netherlands

CoC: 55502105
VAT: NL851741083B01
EORI: NL851741083

Tel: +31 (0)85 002 0881
Email: info@formfutura.com
Website: www.formfutura.com

All information supplied by or on behalf of Formfutura in relation to its products, whether in the nature of data, recommendations or otherwise, is supported by research and, in good faith, believed reliable, but Formfutura assumes no liability and makes no warranties of any kind, express or implied, including, but not limited to, those of title, merchantability, fitness for a particular purpose or non-infringement or any warranty arising from a course of dealing, usage, or trade practice whatsoever in respect of application, processing or use made of the forementioned information or product. The user assumes all responsibility for the use of all information provided and shall verify quality and other properties or any consequence from the use of all such information. Typical values are indicative only and are not to be construed as being binding specifications.
