# Supplementary material for: Tensile characterization and life cycle assessment of fungal-colonized 3D-printed PLA/wood biocomposites
Source: Sci Rep. 2026 Aug 22;16:26365. doi: 10.1038/s41598-026-65923-5 (PMC13499799; doi:10.1038/s41598-026-65923-5)
Supplement: Supplementary file 6 [file 41598_2026_65923_MOESM6_ESM.pdf]

# PLA FILAMENT

POLYLACTID

## Beschreibung:

PLA (Polylactid) Filament ist eines der beliebtesten Materialien für den 3D-Druck. Es handelt sich um umweltfreundliches Material.

## Vorteile:

Umweltfreundlich: PLA-Filament wird aus erneuerbaren Rohstoffen hergestellt und ist biologisch abbaubar. Es hinterlässt einen geringen ökologischen Fußabdruck.

Einfache Handhabung: Es ist leicht zu drucken und erfordert keine beheizte Druckplatte. Es haftet gut auf verschiedenen Druckoberflächen.

Geringer Geruch: Im Vergleich zu einigen anderen Filamenten erzeugt PLA-Filament beim Drucken nur einen geringen Geruch.

## Zusatzinformationen:

Farboptionen: PLA-Filament ist in einer breiten Palette von Farben erhältlich, darunter transparente, opake und glitzernde Varianten.

Kompatibilität: Es ist mit den meisten handelsüblichen 3D-Druckern kompatibel, die das Drucken mit PLA-Filament unterstützen.

Lagerung: PLA-Filament sollte in einem kühlen und trockenen Raum gelagert werden, um Feuchtigkeitsaufnahme zu verhindern.

## Eigenschaften:

| | |
|---|---|
| Dichte | 1,25 $g/cm^3$ |
| Fließspannung | 9,56 g/10min |
| Zugfestigkeit | 69,8 MPa |
| Bruchdehnung | 4,8% |
| E-Modul | 3120 MPa |
| Schlagzähigkeit | 3,4 $kJ/m^2$ |
| Feuchtigkeits-aufnahme | 1968 ppm |
| Schmelz-temperatur | 77 °C – 136 °C |
| Erweichungs-temperatur | 60 °C |
| Glasübergangs-temperatur | 57 °C |

## Druckereinstellungen:

| | |
|---|---|
| Düsen-temperatur | 205 °C ± 10 °C |
| Bett-Temperatur | 0 °C – 60 °C |
| Druckgeschwin-digkeit | 80 mm/min |
| Schichthöhe | >0,08 mm |
| Lüfterstärke | 50-100% |

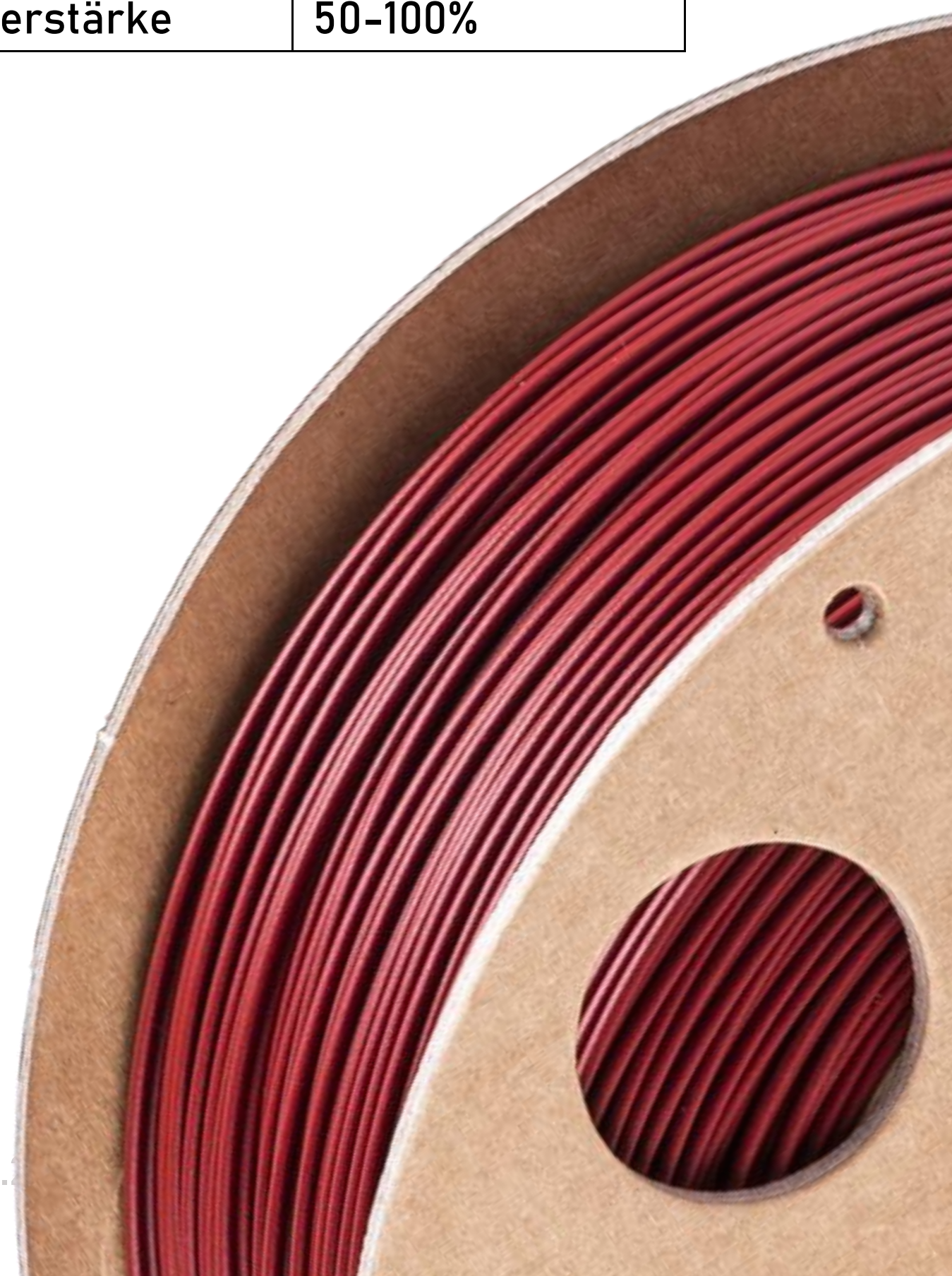

## Farben

- Beige
- Maisgelb
- Elfenbein
- Schwefelgelb
- Gelb
- Gelbgold
- Goldbraun
- Bronzegold
- Hellorange
- Orange
- Gelborange
- Weinrot
- Rot
- Orientrot
- Kupferbraun
- Lila
- Magenta
- Leichtes Pink
- Dunkelblau
- Kobaltblau
- Blau
- Himmelblau
- Leichtes Blau
- Blattgrün
- Dunkelgrün
- Olivgrün
- Apfelgrün
- Leichtes Grün
- Beigebraun
- Braun
- Khaki
- Hellgrau
- Silber
- Grau
- Eisengrau
- Weiß
- Transparent/Natur
- Seidenweiß
- Schwarz
- Glow in the Dark Grün
- Glow in the Dark Blau
- Neon Gelb Transparent
- Neon Grün Transparent
- Neon Orange Transparent
- Neon Pink
- Neon Transparent
